# An Early Stage Researcher's Primer on Systems Medicine Terminology

**DOI:** 10.1089/nsm.2020.0003

**Published:** 2021-02-25

**Authors:** Massimiliano Zanin, Nadim A.A. Aitya, José Basilio, Jan Baumbach, Arriel Benis, Chandan K. Behera, Magda Bucholc, Filippo Castiglione, Ioanna Chouvarda, Blandine Comte, Tien-Tuan Dao, Xuemei Ding, Estelle Pujos-Guillot, Nenad Filipovic, David P. Finn, David H. Glass, Nissim Harel, Tomas Iesmantas, Ilinka Ivanoska, Alok Joshi, Karim Zouaoui Boudjeltia, Badr Kaoui, Daman Kaur, Liam P. Maguire, Paula L. McClean, Niamh McCombe, João Luís de Miranda, Mihnea Alexandru Moisescu, Francesco Pappalardo, Annikka Polster, Girijesh Prasad, Damjana Rozman, Ioan Sacala, Jose M. Sanchez-Bornot, Johannes A. Schmid, Trevor Sharp, Jordi Solé-Casals, Vojtěch Spiwok, George M. Spyrou, Egils Stalidzans, Blaž Stres, Tijana Sustersic, Ioannis Symeonidis, Paolo Tieri, Stephen Todd, Kristel Van Steen, Milena Veneva, Da-Hui Wang, Haiying Wang, Hui Wang, Steven Watterson, KongFatt Wong-Lin, Su Yang, Xin Zou, Harald H.H.W. Schmidt

**Affiliations:** ^1^ Centro de Tecnología Biomédica, Universidad Politécnica de Madrid, Madrid, Spain.; ^2^ Intelligent Systems Research Centre, School of Computing, Engineering and Intelligent Systems, Ulster University, Ulster, United Kingdom.; ^3^ Center for Physiology and Pharmacology, Institute of Vascular Biology and Thrombosis Research, Medical University of Vienna, Vienna, Austria.; ^4^ TUM School of Life Sciences Weihenstephan, Technical University of Munich, Freising, Germany.; ^5^ Faculty of Technology Management, Holon Institute of Technology (HIT), Holon, Israel.; ^6^ CNR National Research Council, IAC Institute for Applied Computing, Rome, Italy.; ^7^ Lab of Computing, Medical Informatics, and Biomedical Imaging Technologies, School of Medicine, Aristotle University of Thessaloniki, Thessaloniki, Greece.; ^8^ Université Clermont Auvergne, INRAE, UNH, Plateforme d'Exploration du Métabolisme, MetaboHUB Clermont, Clermont-Ferrand, France.; ^9^ Biomechanics and Bioengineering Laboratory (UMR CNRS 7338), Université de Technologie de Compiègne, Compiègne, France.; ^10^ Labex MS2T “Control of Technological Systems-of-Systems,” CNRS and Université de Technologie de Compiègne, Compiègne, France.; ^11^ Faculty of Engineering, University of Kragujevac, Kragujevac, Serbia.; ^12^ Bioengineering Research and Development Center (BioIRC), Kragujevac, Serbia.; ^13^ Steinbeis Advanced Risk Technologies Institute doo Kragujevac, Kragujevac, Serbia.; ^14^ Pharmacology and Therapeutics, School of Medicine, Galway Neuroscience Centre, National University of Ireland, Galway, Republic of Ireland.; ^15^ School of Computing, Ulster University, Ulster, United Kingdom.; ^16^ Faculty of Sciences, Holon Institute of Technology (HIT), Holon, Israel.; ^17^ Department of Mathematics and Natural Sciences, Kaunas University of Technology, Kaunas, Lithuania.; ^18^ Faculty of Computer Science and Engineering, Ss. Cyril and Methodius University, Skopje, Macedonia.; ^19^ Laboratory of Experimental Medicine (ULB 222), Medicine Faculty, Université libre de Bruxelles, CHU de Charleroi, Charleroi, Belgium.; ^20^ Northern Ireland Centre for Stratified Medicine, Biomedical Sciences Research Institute, Ulster University, Ulster, United Kingdom.; ^21^ Escola Superior de Tecnologia e Gestão, Instituto Politécnico de Portalegre, Portalegre, Portugal.; ^22^ Centro de Recursos Naturais e Ambiente (CERENA), Instituto Superior Técnico, Universidade de Lisboa, Lisboa, Portugal.; ^23^ Faculty of Automatic Control and Computers, University Politehnica of Bucharest, Bucharest, Romania.; ^24^ Department of Drug Sciences, University of Catania, Catania, Italy.; ^25^ Centre for Molecular Medicine Norway (NCMM), Forskningparken, Oslo, Norway.; ^26^ Centre for Functional Genomics and Bio-Chips, Institute of Biochemistry, Faculty of Medicine, University of Ljubljana, Ljubljana, Slovenia.; ^27^ Department of Pharmacology, University of Oxford, Oxford, United Kingdom.; ^28^ Data and Signal Processing Research Group, University of Vic–Central University of Catalonia, Vic, Spain.; ^29^ Department of Psychiatry, University of Cambridge, Cambridge, United Kingdom.; ^30^ College of Artificial Intelligence, Nankai University, Tianjin, China.; ^31^ Department of Biochemistry and Microbiology, University of Chemistry and Technology, Prague, Czech Republic.; ^32^ The Cyprus School of Molecular Medicine, The Cyprus Institute of Neurology and Genetics, Nicosia, Cyprus.; ^33^ Computational Systems Biology Group, Institute of Microbiology and Biotechnology, University of Latvia, Riga, Latvia.; ^34^ Department of Animal Science, Biotechnical Faculty, University of Ljubljana, Ljubljana, Slovenia.; ^35^ Faculty of Civil and Geodetic Engineering, University of Ljubljana, Ljubljana, Slovenia.; ^36^ Department of Automation, Biocybernetics and Robotics, Jozef Stefan Institute, Ljubljana, Slovenia.; ^37^ Center for Research and Technology Hellas, Hellenic Institute of Transport, Thessaloniki, Greece.; ^38^ Altnagelvin Area Hospital, Western Health and Social Care Trust, Altnagelvin, United Kingdom.; ^39^ BIO3-Systems Genetics, GIGA-R, University of Liege, Liege, Belgium.; ^40^ BIO3-Systems Medicine, Department of Human Genetics, KU Leuven, Leuven, Belgium.; ^41^ Independent Researcher.; ^42^ State Key Laboratory of Cognitive Neuroscience and Learning, and School of Systems Science, Beijing Normal University, Beijing, China.; ^43^ Northern Ireland Centre for Stratified Medicine, Ulster University, Londonderry, United Kingdom.; ^44^ Shanghai Centre for Systems Biomedicine, Key Laboratory of Systems Biomedicine (Ministry of Education), Shanghai Jiao Tong University, Shanghai, China.; ^45^ Faculty of Health, Medicine & Life Science, Maastricht University, Maastricht, The Netherlands.

**Keywords:** systems medicine, multiscale modeling, multiscale data science

## Abstract

**Background:** Systems Medicine is a novel approach to medicine, that is, an interdisciplinary field that considers the human body as a system, composed of multiple parts and of complex relationships at multiple levels, and further integrated into an environment. Exploring Systems Medicine implies understanding and combining concepts coming from diametral different fields, including medicine, biology, statistics, modeling and simulation, and data science. Such heterogeneity leads to semantic issues, which may slow down implementation and fruitful interaction between these highly diverse fields.

**Methods:** In this review, we collect and explain more than100 terms related to Systems Medicine. These include both modeling and data science terms and basic systems medicine terms, along with some synthetic definitions, examples of applications, and lists of relevant references.

**Results:** This glossary aims at being a first aid kit for the Systems Medicine researcher facing an unfamiliar term, where he/she can get a first understanding of them, and, more importantly, examples and references for digging into the topic.

## Introduction

Although death has always been the end of every human's life, mankind has been trying to delay that as much as possible. It is, thus, not surprising that one of the most ancient forms of science, if not the first, has been medicine, starting with documents going back to ancient Egypt and Greece.^1^ In the previous century, technical advances (from vaccines to genome sequencing) have supposed a revolution in medicine, and have allowed a substantial reduction in mortality rates. However, this trend is now experiencing diminishing returns: New drugs are nowadays being developed less frequently and at a higher cost; they are beneficial to smaller subsets of the population, and consequently have less impact on life expectancy. In parallel, mankind has recently witnessed an Information Technology (IT) revolution, in which data are gathered and processed at unprecedented rates, given birth to applications that would have appeared as science fiction as recently as 20 years ago. Following the theory of Kondratiev waves,^2^ postulating the existence of waves of 40–60 years with high sectoral growth, could it be that the next wave will have medicine at its focus, and specifically through the merging of both revolutions?

Such merging is actually taking the form of the so-called Systems Medicine, an interdisciplinary field of study that looks at the human body as a system, composed of interacting parts, and further integrated into an environment.^3,4^ It considers that these complex relationships exist on multiple levels, and that they have to be understood in light of a patient's genomics, behavior, and environment. The analysis of a disease then starts with real data, coming from a large number of patients (thus to ensure that the natural variability is taken into account) and covering all aspect of them, from genetics to the environment. Machine-learning and mathematical models are then developed, aimed at finding the most efficient way of disrupting the disease in a specific patient.

Even after this oversimplified description, it is clear that systems medicine requires skills and knowledge not considered in standard medical curricula, or alternatively the collaboration between researchers of different backgrounds. The revolutionary idea behind systems medicine is, thus, responsible for its main drawback: the need for understanding and combining concepts coming from diametral different fields, including statistics, modeling and simulation, and data science.^5^ The researcher wanting to enter this world will face an additional problem: Although a large number of books and papers can be found on, for example, data-mining concepts, these are usually not written with a medical practitioner in mind. Not just the required background, but even the basic terminology can become a major barrier.

This review addresses the semantic issues this implies, which may slow down implementation and fruitful interaction between these highly diverse fields, by providing the first version of the Systems Medicine Dictionary.^*^ Specifically, the practitioner coming from medicine will find in it a large number of modeling and data science terms, along with some synthetic (although comprehensive) definitions and a list of relevant references. Similarly, a researcher with a background in modeling and data will here find an explanation of the basic systems medicine terms. It is worth noting that these definitions are not exhaustive, as both their selection and the corresponding content have been guided by the personal view of the authors. In addition, some terms described here represent fields of research on their own, whose characterization can hardly be contained in a monographic book. This work, thus, represents the first aid kit for the systems medicine researchers facing an unfamiliar term. They will here get a first understanding of it; and, more importantly, examples and references for digging into the topic.

Science, in general, and medicine, in particular, can benefit from approaches that are different from what was done earlier, as these can have multiplicative effects on knowledge and understanding in general; this may lead to new insights and ideas for new hypotheses, and eventually to breakthroughs unattainable via the old and tested ways of thinking and acting. In turn, this requires crossing discipline boundaries and provides new angles to old information. We expect this glossary to be especially useful to the younger readership, for example, PhD students and early career researchers, as they are at a better position to break away from old conventionalisms while significantly boosting their careers.

## Concepts from Systems Medicine, Modeling, and Data Science

All terms are included here in alphabetical order, and they are further listed in Table 1. Table 2 also reports a list of the acronyms that appear in the text, and the corresponding meaning. Finally, underlined words, for example, agent-based modeling (ABM), refer to terms that are defined here.

**Table 1.** List of the terms described here

Agent-based modeling	Artificial neural networks	Bayesian filtering
Bayesian networks	Bayesian smoothing	Bayesian statistics
Biofluid mechanics	Bioheat transfer	Biological networks
Biomaterials	Biomechanics	Cellular automata
Clinical decision support systems	Clustering	Complex networks
Complex systems	Computational drug repurposing	Constraints
Context awareness systems	Correlation networks	CRISP-DM
Cross-validation	Data analysis software	Data fusion and data integration
Data mining	Decision Tree	Decision support systems
Deep learning	Digital Health	Digital Twin
Dissipative particle dynamics	Erdős–Rényi model	Exposome
FAIR principles	Feature selection	Finite element method
Finite volume method	Frequentist statistics	Functional networks
Gene set enrichment analysis	Granger causality	Graph embedding
Hidden conditional random fields	Imputation	*In silico* modeling
Integrative analysis	Interactome	Internet of things
Lattice Boltzmann method	Machine learning	Mediation analysis
Medical informatics	metaboAnalyst	Metabolomics
Model robustness	Model verification and validation	Morphometric similarity networks
Multiphysics systems	Multilayer networks	Multiscale biomolecular simulations
Multiscale modeling	Network analysis software	networkAnalyst
Network medicine	Null models	Nvidia Clara
Object-oriented modeling	Ontologies	Parameter estimation
Parameter identifiability	Parameter sensitivity analysis and uncertainty quantification	Permutation test
Phase transition	Physiome	Precision medicine
Probabilistic risk analysis	Quantitative systems pharmacology	Random forest
Random graphs	Scale-free networks	Simulated annealing
Small-world network	Smoothed-particle hydrodynamics	Solid–fluid interaction
Statistical bioinformatics	Statistical networks	Support vector machine
Surrogate model	Systems biology	Systems bioinformatics
Systems dynamics	Systems engineering	Systems medicine
System of systems	Standards	Structural covariance networks
Time-evolving networks	Time-scale separation	Variation partitioning
Virtual physiological human		

CRISP-DM, Cross-Industry Standard Process for Data Mining; FAIR, Findability, Accessibility, Interoperability, and Reusability.

**Table 2.** List and explanation of the acronyms used throughout the review

2SSP	Two-Stage Stochastic Programming
AAL	Ambient-assisted living
ABM	Agent-based modeling
AI	Artificial intelligence
ANN	Artificial neural networks
BI	Business intelligence
BIC	Bayes information criteria
BPPV	Benign paroxysmal positional vertigo
CA	Cellular automata
CDSS	Clinical decision support system
CFD	Computational fluid dynamics
DDA	Drug–disease association
DDI	Drug–drug interaction
DPD	Dissipative particle dynamics
DSS	Decision support system
DT	Decision tree
EEG	Electro-encephalography
FBA	Flux balance analysis
FEA	Finite element analysis
FEM	Finite element method
fMRI	Functional magnetic resonance imaging
FVM	Finite volume method
GCN	Gene co-expression network
GRN	Gene-regulatory network
GSEA	Gene set enrichment analysis
HCRF	Hidden conditional random fields
HMS	Health care monitoring system
HSH	Health smart homes
ICT	Information and communication technologies
IoMT	Internet of medical things
IoT	Internet of things
IT	Information technology
LB	Lattice Boltzmann
LDL	Low-density lipoprotein
MEG	Magneto-encephalography
MFA	Metabolic flux analysis
MICE	Multiple imputation by chained equations
MMS	Multiscale modeling and simulation
MSC	Multiscale computing
NLP	Natural language processing
PaaS	Platform as a service
PCA	Principal-component analysis
PIN	Protein interaction network
PK/PD	Pharmacokinetic/pharmacodynamic
PPI	Protein–protein interaction
PRA	Probabilistic risk analysis
QM/MM	Quantum mechanical and molecular mechanical
QSP	Quantitative systems pharmacology
RF	Random forest
RFE	Recursive feature elimination
RSM	Response surface models
SA	Simulated annealing
SDK	Software Development Kit
SPH	Smoothed-particle hydrodynamics
TF	Transcription factor
t-SNE	t-Distributed stochastic neighbor embedding
UPR	Unfolded protein response

### Agent-based modeling

ABM (also known as Individual-based modeling, Multi-agent Systems, and Multi-agent autonomous Systems) is a modeling/simulation paradigm that is especially suited for studying complex systems, that is, systems composed of a large number of heterogeneous interacting entities, with each having many degrees of freedom. A very open definition of this mathematical discrete modeling paradigm is to represent a physical or biological system on the basis of entities (called agents) with defined properties and behavioral rules, and then to simulate them in a computer to reproduce the real phenomena and to perform what-if analysis.^6^ Agents have, thus, to be understood as autonomous entities, each one with an internal state representing its knowledge about the environment, and rules (or algorithms) to interact with other agents. This broad definition can then encompass from simple particles to autonomous software with learning capabilities. To illustrate, these can be from “helper” agents for web retrieval,^7,8^ robotic agents to explore inhospitable environments,^9^ up to lymphocytes in an immune system reaction simulation.^10–12^ Roughly speaking, an entity is an “agent” if it is distinguishable from its environment by some kind of spatial, temporal, or functional attribute: An agent must be identifiable. In addition, agents can be identified on the basis of four basic properties: autonomy, that is, the behavior of each agent is not guided by rules defined at a higher tier; social ability, that is, their capacity of interacting with other agents; reactivity, in that they react to perceived changes in the environment; and pro-activeness, that is, the ability to take the initiative. Moreover, it is also conceptually important to define what the agent “environment” in an ABM is. This can be implicitly embedded in the behavioral rules or be explicitly represented as a different “modeled object” with a well-defined set of characteristics that influence the agent's behavior.

An ABM simulation may start from simple agents, locally interacting with simple rules of behavior, responding to perceived environmental cues and trying to achieve a local goal. However, the simplicity of the composing elements does not derive in the simplicity of the overall dynamics. From this simple configuration, a synergy may emerge, which leads to a higher-level whole with much more intricate behavior than the component agents (holism, meaning all, entire, total).

If the first examples of agent-based models were developed in the late 1940s, only computers could really show their modeling power. These include the Von Neumann machine, a theoretical machine capable of reproduction,^13^ that is, of producing an identical copy of itself by following a set of instructions. This idea was then improved by Ulam,^14^ by suggesting machines to be built on paper, as collections of cells on a grid. This idea inspired von Neumann to create the first of the models later termed cellular automata (CA). Building on top of these, John Conway constructed the well-known “Game of Life,” a simple set of rules that allow evolving a virtual world in the form of a two-dimensional checkerboard, and which has become a paradigmatic example of the emergence of order in nature. How do systems self-organize themselves and spontaneously achieve a higher-ordered state? These and other questions have been addressed in-depth in the first workshop on Artificial Life (ALife) held in the late 1980s in Santa Fe. This workshop shaped the ALife field of research,^15^ in which ABM models are the main form of modeling and simulation.

The ABM proved very successful in theoretical biology. In this specific research domain, ABM is emerging as the best modeling paradigm that is able to accommodate the need to represent more than one level of space-time description, thus fitting the multiscale specification. Beyond the aforementioned works on the immune system, examples include cancer modeling,^16,17^ or epidemics predictions.^18,19^ For further discussions and examples, the reader may refer to An et al.^20^

### Artificial neural networks

**FIG. 1.**
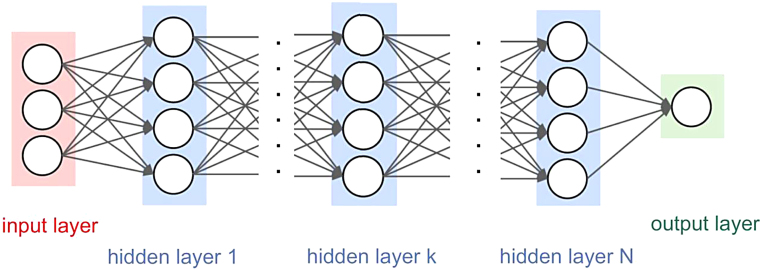
Graphical representation of ANN. ANN, artificial neural network.

Artificial neural networks (ANN) are inspired by the neural networks that exist in mammal brains.^21^ They represent a programming paradigm that helps a computer to process complex information to learn from the observational data. The network itself consists of connected units or nodes called artificial neurons (based on neurons in a biological brain) that are organized in layers. The first layer is called the input layer and is connected to the input signals. The input layer is followed by one or more hidden layers, all the way to the output layer connected to the output signals. Analogous to the synapses in a biological brain, signals are transmitted from one neuron to another. The output of one artificial neuron is computed when a nonlinear function is applied on the sum of its inputs. Usually, the weights and biases are added to adjust the learning process. Weights increase or decrease the strength of the signal at a connection, and biases represent the threshold to delay the triggering of the activation function. Mathematically, this can be represented as (Fig. 1):

$$Output=f\left(\sum weight*input+bias\right).$$

For ANN to learn from the provided data, they need to have a huge amount of information used as a training set. During the training period, the ANNs output is compared to the human-provided description of what should be observed (called *target*). If they are the same, weights are validated, and in case of incorrect classification, its learning will be adjusted.^22^ In the end, an unknown signal (not used in the training set) will be used as the input, and we expect the network to correctly predict the output (this process is called *generalisation*). As an example, in the process of classification of images as images with a dog or cat, the training set would be thousands of images already classified as dog or cat image. After the training, the ANN should be able to classify future images based on the trained model.

Although ANNs were originally aimed at solving specific biology problems, over time their application extended to a wide spectrum of tasks, including systems medicine through genomics, drug repurposing, or personalized medicine. Not surprisingly, many reviews are available. For instance, Awwalu et al. investigated the adequacy of using ANN, among other artificial intelligence (AI) algorithms, in solving personalized medicine and precision medicine problems.^23^ Ching et al. have developed an ANN framework called Cox-nnet to predict patient prognosis from high-throughput transcriptomics data.^24^ Bica et al. have introduced a novel neural network architecture for exploring and integrating modalities in omics datasets, especially in cases where a limited number of training examples was available.^25^ Also, some examples of application of deep neural networks could be found in using neural networks to learn an embedding that substantially denoises expression data, making replicates of the same compound more similar.^26^ Donner et al. used ANNs to identify drugs with shared therapeutic and biological targets, even for compounds with structural dissimilarity, revealing functional relationships between compounds and making a step forward toward the drug repurposing based on expression data.^26^

### Bayesian filtering

A class of methods that allows estimating the current state, that is, the value of the observed variable(s), based on noisy measurements of the current and previous states. For instance, the spread of infectious diseases could be modeled with the help of Bayesian filters, where the time-varying variables are, for example, estimations of the number of susceptible, infected, healed, and dead individuals taken in the current and some previous time moments.^27^ For more information, see Särkkä.^28^

### Bayesian networks

Bayesian networks (also known as Bayes networks, belief networks, Bayes/Bayesian models, and probabilistic directed acyclic graphical models) are a type of directed graphical model (i.e., a graph expressing the conditional dependencies between variables) that combines graph theory and probability theory (see also the Bayesian Statistics section). They present a formalism designed to address problems involving uncertainty and complexity. The Bayesian network approach can be seen as both a statistical and an AI-like knowledge-representation formalism. It is a useful tool for describing mechanisms involving stochasticity, cohort heterogeneity, and knowledge gaps, which are common features of medical problems, and has been utilized for diagnosis, treatment selection, and prognosis^29^ as well as for analyzing probabilistic cause–effect relationships (i.e., estimating the likelihood of a set of factors to be contributing to an observation, e.g., the relationship between symptoms and potential underlying mechanisms). Bayesian networks are constructed as directed acyclic graphs, where nodes represent unique variables that have a finite set of mutually exclusive states, whereas edges represent conditional dependence and the absence of edges conditional independence.^30^ For each variable *A* with parents $B_{1},B_{2},\ldots ,B_{n}$, there is a conditional probability table *P* given as $P\left(A|B_{1},B_{2},\ldots ,B_{n}\right)$.^30^ Importantly, Bayesian networks satisfy the local Markov property, meaning that nodes are conditionally independent of its nondescendants given its respective parents. This characteristic permits a simplification of joint distributions within the model, allowing for efficient computation. In the most simple approach, a Bayesian network is specified by using expert knowledge; in the case of complex interactions, the network structure and parameters need to be learned from data.

#### Inference and learning in Bayesian networks

Given probability tables of the variables in a Bayesian network and conditional independencies, joint probability distributions can be calculated and utilized to infer information within the network and for structural learning. This approach can be used for different probabilistic inference methods, for example, for estimating the distribution of subsets of unobserved variables given observed variables (so-called evidence variables). Further, Bayesian networks can be utilized to express causal relationships and combine domain knowledge with data, and, importantly, can thus be used for probabilistic parameter estimation.

Examples of the use of Bayesian networks in medicine include the diagnosis and prediction of disease trajectory,^31–33^ health care planning,^34,35^ and molecular data analysis.^36^ Although this is a popular and successful option for modeling in the medical domain, they should be used with caution in complex problems with multiple feedback loos and closed-loop conditions.

#### Most relevant limitations

Bayesian networks commonly rely on prior knowledge/belief for construction and inference; thus, the quality and usefulness of a respective network is directly dependent on the usefulness and reliability of this prior knowledge. In the case of expert-constructed networks, it may further be challenging to translate this knowledge into probability distributions. Bayesian networks are constructed as acyclic graphs and thus do not support the implementation of feedback-loops,^37^ although this may be addressed by using dynamic Bayesian networks.^38^ Bayesian networks have limited ability to deal with continuous variables, a limitation most commonly addressed by discretizing these variables, which, in turn, has tradeoffs.^39^ Lastly, Bayesian learning and inference can become very computationally expensive, to the point that a network becomes impossible to compute and the search space needs to be reduced by using different heuristics.^40,41^

### Bayesian smoothing

This is a class of methods for reconstructing previous state(s), having noisy measurements of the current and the previous states. Brain imaging is an example of an area that can take advantage of the Bayesian filters and smoothers relying on sensor measurements of different values.^28^

### Bayesian statistics

Bayesian statistics is a Bayesian interpretation of probability in which probability expresses a degree of belief in an event, as opposed to a fixed value based on frequency—see the Frequentist Statistics section.

The basic framework of Bayesian analysis is quite straightforward. Prior distributions are associated with parameters of interest to represent our initial beliefs about them, for example, based on objective evidence, subjective judgment, or a combination of both. Evidence provided by further data is summarized by a likelihood function, and the normalized product of prior and the likelihood forms a posterior distribution. This posterior distribution contains all the currently available information about the model parameters. Note that this is different from the standard frequentist approach, and that both methods do not always give the same answers; and this is fueling an ongoing debate between proponents of both approaches.^42–44^ At the same time, the use of a Bayesian approach yields results that go beyond what are obtainable through a frequentist perspective.^45–47^ In what follows, the most important points of Bayesian and frequentists disagreements and differences are discussed: prior distributions, sequential analysis, and confidence intervals.

#### The (subjective) choice of prior distribution

The specification of prior distribution is a matter of ongoing concern for those contemplating the use of Bayesian methods in medical research.^48^ It is not without a reason that frequentists object to this concept. Any conclusions drawn from the posterior distribution will be impacted by this choice. If the prior distribution is informative, that is, already carries strong evidence for certain values of unknown parameters, then new data might have no significant impact at all (which is not a bad thing if our prior distribution reflects the truth). Many authors devoted their thoughts to the formalization of the prior distribution selection,^49–52^ and they all have made suggestions regarding the elicitation and quantification of prior opinions of clinicians. However, it is still a very difficult task. Even minor mistakes in the prior elicitation can propagate to significant errors in the posterior inferences. The subjectivity in the elicitation of expert opinions is the main critique of the Bayesian approach. Actually, in very complex problems, such elicitation might even be impossible to many parameters. However, uninformative priors, the kind that also have a claim to objectivity, are the Bayesian response.^53^ In fact, there is a strong movement toward objective uninformative priors in the Bayesian community.

This struggle to develop the objective Bayesian framework produced quite many different approaches on how to devise objective prior distribution. The most famous of these is the Jeffreys-rule prior.^54^ Reference priors^55,56^ are a refinement of the Jeffreys-rule priors for higher dimensional problems and have proven to be remarkably successful from both Bayesian and non-Bayesian perspectives. Maximum entropy priors^57^ are another well-known type of noninformative prior, although they often also reflect certain informative features of the system being analyzed. Invariance priors, as mentioned earlier, matching priors,^58^ and admissible priors^59^ are other approaches being extensively studied today. Methods on how to select a prior distribution from this vast universe of possible distributions are discussed in Kass and Wasserman.^60^ Caution is advised when considering a noninformative distribution. Sensitivity analysis should always be performed, because in small sample cases, noninformative prior distribution can still influence the posterior results.^61^ On the other hand, arbitrariness is not so unfamiliar to frequentists' practices as well.

#### Sequential analysis

The Bayesian approach includes a generally accepted stopping rule principle: Once the data have been observed, the reasons for stopping the experiment should have no effect on the evidence reported about unknown model parameters. Frequentists' practice, on the other hand, is different. If there are to be interim analysis during the clinical trial, with the option for stopping the trial early should the data look convincing, frequentists feel that it is mandatory to adjust allowed error probability (down) to account for the multiple analysis.^42^

Stopping rules are especially important in clinical trials, and Bayesians pick up on this theme as early as 1992, with four seminal papers on colorectal cancer clinical trials.^62–66^ Currently, Bayesian stopping rules are being used in all phases of trials—see Ashby^46^ for a complete review. In fact, the increasing use of Bayesian statistical methods in clinical research is supported by their capacity to adapt to information that is gathered during a trial, potentially allowing for smaller, but yet more informative trials, and for patients to receive better treatment.^67^

#### Confidence intervals

The concept of confidence intervals is purely frequentists. However, the way it is (wrongly) interpreted is Bayesian. Confidence interval represents the precision of a parameter estimate as the size of an interval of values that necessarily include estimate itself. A true understanding of the concept would look like this: If new data were to be repeatedly sampled, the same analysis carried out, and a series of 95% confidence intervals calculated, 19 out of 20 of such intervals would, in the long run, include the true value of the quantity being estimated.^68^ However, many researchers (mistakenly and fundamentally incorrect) interpret this interval as a 0.95 probability that the true parameter is in the interval. If one would be truly Bayesian from the beginning of the analysis, Bayesian credible intervals^69^ would be considered as exactly the probability that the unknown parameter is contained in it. In fact, in certain prior distribution cases, Bayesian credible intervals are exactly the confidence intervals, only the interpretation is different.

#### The interplay of Bayesian and frequentist analysis

Currently, there is a trend of using notions from one type of approach to support analysis of another approach. Of many topics, several should be mentioned in this brief note: empirical Bayesian analysis, where prior distribution is estimated from the data^70^; approximate model selection methods, such as BIC,^71^ similar to the usage of Akaike information criteria; robust Bayesian analysis,^72^ which recognize the impossibility of complete subjective specification of the model and prior distribution, etc. From the frequentist theory viewpoint, the most convincing argument in favor of the Bayesian approach is that it intersects widely with the three notions of classical optimality, namely, minimaxity, admissibility, and equivariance.^73^

### Biofluid mechanics

Biofluid mechanics is the application of principles of fluid mechanics on the dynamics of motion of biofluids inside and around of living organisms and cells.^74^ The main applications of biofluid dynamics are the study of the circulatory system with the blood-flow inside vessels of various sizes, the study of the respiratory system with the air-flow inside the lungs, and also the lubrication of synovial joints.^75^ The study of biofluid dynamics has allowed many therapeutic applications such as artificial heart valves,^76^ stents, and in the future artificial lungs.^77^ Biofluid dynamics can be studied with simulations and experiments. Computational fluid dynamics simulations can be used to better understand the flow phenomena of the biofluids inside the complex geometry of vessels. Biofluid dynamics can also be studied with *in vivo* experiments, with the use of noninvasive medical imaging methods such as Doppler ultrasound and magnetic resonance imaging (MRI), invasive methods such as angiography but also with more straightforward methods as the pressure cuff used to measure blood pressure.^78^

### Bioheat transfer

Bioheat transfer concerns the rate of heat transfer between a biological system and its environment. The main difference regarding heat transfer of biological systems to nonbiological ones is the blood perfusion through the extended network of vasculature in biological systems that directly affects the local temperature of the living tissue.^79^ The main research subjects of bioheat transfer are the thermal interaction between the vasculature and tissue, tissue thermal parameter estimation,^80^ human thermal comfort, thermoregulation, safety of heat transfer to living tissue due to microwave, ultrasound or laser exposure due to environmental exposure or for therapeutic applications.^81^ Because biochemical processes are governed by local temperature, bioheat transfer also plays a major role in the rate of these processes.

### Biological networks

The concept of complex networks represents a powerful tool for the representation and the analysis of complex systems, and especially to describe their internal interaction structure. Recently, the so-called network biology approach^82^ has been fruitfully applied in many different biological areas, from gene regulation, to protein–protein interactions (PPIs), to neural signals,^83^ to finally hit clinical applications: Network medicine is today at the forefront of modern quantitative approaches in medical sciences.^84^ Here, with no claim of exhaustiveness, we list the main types of biological networks.

#### PPI networks

PPIs are physical contacts, stable or transitory, between two or more proteins created by electrostatic forces between the so-called protein surfaces, that is, the “exposed” regions of the three-dimensional structures of folded proteins. These contacts are at the base of most biological functions, as, for instance, of signal transduction, cell metabolism, membrane transport, or muscle contraction. It is, thus, clear that the analysis of how proteins interact between each other is essential to understand cellular processes in healthy and in pathological conditions. Sets of proteins and their interactions are generally referred to as protein interaction networks (PINs), mathematically represented by undirected graphs. The specific analyses performed on PINs depends on the overall goal of the study; to illustrate, one may try to identify the most prominent element for a given function (e.g., gene target prioritization),^85^ or the set of lethal proteins in a cell.^86^ Methods for the detection of protein interaction encompass experimental (e.g., yeast-two-hybrids, mass spectrometry) or *in silico* (ortholog-based) approaches.^87,88^

#### Gene-regulatory networks

Gene-regulatory networks (GRNs) are networks of causative and regulative interactions (biochemical processes such as reactions, transformations, interactions, activations, inhibitions: the links) between transcription factors (TFs) and downstream genes (the nodes), represented with directed graphs and inferred by gene expression data.

Methods to extrapolate GRNs are based on information-theoretic criteria, co-expression metrics, or regression approaches, among others. For example, the mutual information (MI) approach is often used, that is, a dimensionless metric that states how much the knowledge of a random variable tells about another one. A value of MI of zero indicates that the two variables are completely independent; on the other hand, MI >0 implies that they are connected, as knowing one of them is equivalent to (partially) knowing the other. Thus, if MI >0 for the expression of two genes, we can infer that one of them is (partially, at least) driving the other.^89^

Though created in an indirect way, inferred GRNs aim at representing real physical, directed, and quantitatively determined interaction events, both between genes and, and between them and their products. The final aim is the discovery of key functional relationships between RNA expression and chemotherapeutic susceptibility.^90^ Recently, data from single-cell gene expression have become mature and have been approached by using partial information decomposition to detect putative functional associations and to formulate systematic hypotheses.^91,92^

Validation of GRNs has traditionally been performed in two ways. On the one hand, one can resort to “gold standards,” that is, sets of interactions that have been validated; on the other hand, one can observe the biological system under study *in vitro*, by inducing a perturbation and by observing whether the real and predicted effects coincide.^93,94^

#### Gene co-expression networks

Gene co-expression networks (GCNs) are basically RNA transcript–RNA transcript association networks: Nodes of the network correspond to genes, which are pairwise connected when an appreciable transcript co-expression association between them exists. Networks are then calculated by estimating some kind of similarity score from expression data and by applying a significance threshold; the result is usually a undirected graph. In reconstructing GCNs, normalization methods, co-expression correlation (e.g., Pearson's or Spearman's correlation measures), significance, and relevance estimation are calculated. Graphical Gaussian Models (e.g., “concentration graph” or “covariance selection” models) are also used, along with edge removal based on gene triplets analysis (e.g., the ARACNE tool), regression methods, and Bayesian networks.^95^

#### Signaling networks

Signaling pathways are cascades of molecular/chemical interactions and modifications to carry signals from cell membrane receptors to the nucleus to arrange proper biological responses to stimuli, on human or microbial levels. The process of reconstructing signaling networks has typically been based on gene knockout techniques, which are effective in describing cascades in a linear or branched manner. Nevertheless, recent screens suggest a switch from such cascades to networks with complex interdependencies and feedbacks,^96^ which require methods that are able to infer aspects and features of signaling processes from high-throughput -omic data in a faster and systemic way. In general, such inference problems can be reduced to the definition of suitable optimal connected subgraphs of a network originally defined by the available data; examples include the Steiner tree approaches (based on the shortest total lengths of paths of interacting proteins), linear programming, and maximum-likelihood (e.g., tagging proteins as activators or repressors to explain the maximum number of observed gene knockout). Alternatives include the use of a probabilistic network, for example, network flow optimization (Bayesian weighting schemes for underlying PPI networks coupled with other -omics data), network propagation (gene prioritization function that scores the strength-of-association of proteins with a given disease), or information flow analysis (based on the identification of proteins dominant in the communication of biological information across the network).^97,98^

#### Metabolic networks

Metabolic network reconstruction is generally referred to as the annotation process of genes and metabolites for the determination of the metabolic network's elements, relationships, structure, and dynamics.^83^ It can be identified on human, microbial and their joint co-metabolic levels. It is usually possible to infer the enzymatic function of individual proteins, or to reconstruct larger (or even whole) metabolic networks. Techniques such as metabolic flux analysis (MFA) and its improvements (e.g., isotopically nonstationary MFA), and flux balance analysis have become largely utilized for the predictions of concurrent fluxes of multiple reactions. Recently, computational approaches coupling MFA with mass spectrometry have been also implemented. Single-enzyme function prediction can be carried out by resorting to machine learning, especially when the enzyme does not show significant similarity to existing proteins; or to “annotation transfer” approaches, based on the use of reference databases or orthologs to tag specific DNA sequences. Comparative pathway prediction methods use established functional annotations to check for the existence of new reactions, whereas explorative pathway prediction techniques (not using existing annotations) can be graph-theoretic (e.g., by weighting paths of metabolite connectivity) or constraint-based (e.g., elementary mode analysis), or both.^99,100^

#### TF networks

When talking about disease and transformation from health to disease, we cannot avoid the TF networks that were enabled by technological advances, such as genome-wide large-scale analyses, genome editing, single-cell analyses, live-cell imaging, etc. Enhancer locations and target genes are keys to TF network models.^101^ The original definition of enhancers is that they represent functional DNA sequences that can activate (enhance) the rate of transcription from a heterologous promoter, independent of their location and orientation.^102^ Determining the function of enhancers and whether TFs bind to them was accelerated by the CRISPR/Cas9 and other genome-editing technologies, as well as by the data collected within the large-scale efforts, such as the Human Epigenome, ENCODE, etc. If we combine the experimental evidence of TFs binding to specific promoter or enhancer DNA elements, at specific genomic loci, we can construct TF network models and maps, to predict biological behavior *in silico* and further guide experimental research. In principle, the TF network models are simple, consisting of subnetworks with nodes (genes and proteins) and edges that link the TFs to their functional targets. More complex models can, nevertheless, be used, for instance integrating Boolean and Bayesian approaches—see Brent^101^ for a review.

The TFs work predominantly in a tissue-specific manner to define the cell phenotypes. For a maximal output, different TFs usually cooperate and synergize, to modulate changes in gene expression.^103^ A TF network map is a graph where we can see which TFs directly regulate a gene by binding to one of its promoter or enhancer elements. A TF network map includes the basic biochemical knowledge, similarly as the metabolic network map. It links the TFs with target genes, taking into account the proper physiological or patophysiological conditions and signals (endogenous and external), as well as the context of the time (development, aging, circadian, etc.). Several approaches have been developed to model and/or graphically represent the TF networks, such as the PetriNets^104^ and the ARACNE algorithm that has been recently upgraded to suit also the single-cell gene expression data.^105^ The NetProphet 2.0^106^ is another algorithm for TF network mapping that can as accurately as possible identify TF targets. Another representation of TF networks are the maps that are built directly from transcriptome data by applying the enrichment procedures. These maps show whether the expression of individual TFs is related. For example, the KEGG pathways^107^ and TRANSFAC database were used for functional enrichment studies.^108^ Gene sets containing more than five elements were constructed and tested for enrichment by using the *PGSEA* package, and the TFs were merged based on their ID irrespective of their binding sites. In this manner, the TF enrichment analyses confirmed an increased unfolded protein response and metabolic decline after depleting one of the genes from cholesterol synthesis in the liver.^109^

### Biomaterials

Biomaterial is a synthetic material that is used to replace part of a living system or to function in intimate contact with living tissue.^110,111^ Although there are different definitions of a biomaterial, the Clemson University Advisory Board for Biomaterials has officially defined a biomaterial as “a systemically and pharmacologically inert substance designed for implantation within or incorporation with living systems.” One must differ biomaterial from biological material (i.e., bone matrix or tooth enamel), which is produced by a biological system. Other materials that should be differentiated are artificial materials that are simply in contact with the skin (i.e., hearing aids and wearable artificial limbs), which are not biomaterials since the skin acts as a barrier with the external world. The main applications of biomaterials include assistance in healing, to improve function and correct abnormalities or replacement of a body part that has lost function due to disease or trauma. Advances in many fields, including surgery, have permitted materials to be used in many cases and wider scope.^112,113^

### Biomechanics

Biomechanics is the application of classical mechanics to the study of biological systems. Laws of physics for statics, kinematics, dynamics, continuum mechanics, and tribology are applied for the study of biological systems from a single cell to whole human bodies.^114^ Biomechanics studies are employing both experiments and numerical simulations. Experiments in biomechanics are performed both *in vitro* and *in vivo*. Common experiments include measurements of kinematics and dynamics of human motion (gait analysis),^115,116^ soft tissue deformation and impact studies (tension-compression tests, impact tests, three-point bending tests),^117^ electromyography for neuromuscular control,^118^ but also experiments at microscopic level with dynamic loading of cells with microscopic cantilevers setups.^119^ Simulation of biomechanics systems has allowed the testing of conditions that would be dangerous to test with human participants or biological tissue, with applications ranging from vehicle safety with simulated crash tests using active human body models, study of biological systems with complex geometries that is not possible to measure their deformation response with experiments, as brain deformation during head impacts and faster and easier-to-perform parametric studies. However, it is important when using a simulation model to consider the range of parameters for which the model is valid.

### Cellular automata

The CA are defined as abstract and discrete (spatially and temporally) computational systems that showed its application as general models of complexity and as more specific representations of nonlinear dynamics in a variety of scientific fields. The CA are composed of a finite (countable) set of homogeneous and simple units, called *atoms* or *cells*. These cells have an internal status that can take a finite set of values, and that is updated at each time step through functions or dynamical transition rules—generally as a function of the states of cells in the local neighborhood. It should be mentioned that CA are abstract, meaning they can be specified in purely mathematical terms and physical structures can implement them. Since CA are computational systems, they can compute functions and solve algorithmic problems, therefore displaying complex emergent behavior. Because of that, they are attracting a growing number of researchers from the cognitive and natural sciences interested in pattern formation and complexity in abstract setting.^120^ The CA have also been applied to some medical problems, as, for instance, image segmentation^121,122^ or infection modeling.^123–125^

### Clinical decision support systems

Clinical decision making involves clinicians making decisions about patient diagnosis and treatment.^126^ Clinical decision making has traditionally largely been determined by human expertise. As of now, clinicians still make the final decisions on weighing across evidence, for example, from clinical data records.

Various statistical and mathematical methods,^127^ and knowledge-based approaches using dictionary-defined knowledge (e.g., with “if-then” rules)^128^ have now been used to aid clinical decision making, resulting in more quantitative, standardized, accurate, and objective decisions. This has led to the development of medical or clinical decision support systems (CDSSs), often in the form of computer software or health technology, aiding human experts with interpretation, diagnosis, and treatment.^129^

The rise of AI, particularly machine learning, has led to another form of CDSSs that is “non-knowledge-based.” Some of these approaches, for example, deep-learning algorithms, have been claimed to outperform human experts in diagnosis of specific illness.^130^ However, interpretability or explainability of the results of such approaches hinder their use in practice.^131^ It should be noted that CDSSs still remain not as highly adopted by users, perhaps partially due to general lack of engagement from clinicians, physicians, or health specialists.^132^

### Clustering

In data mining, any problem involving the division of data into groups (clusters), such that each one of them contains similar records (according to some similarity measures), and that dissimilar records are organized into different clusters. It is also called *unsupervised learning*, as no *a priori* information about the structure of the groups is used. An alternative definition of clustering is proposed in Ref.^133^: “partition a given data set in groups, called clusters, so that the points belonging to a cluster are more similar to each other than the rest of the items belonging to other clusters.”

Although consensus on a unique classification of clustering algorithms has not been achieved, it is customary to divide such algorithms according to their underlying hypothesis^134^:

- Hierarchical-based. Hierarchical clustering combines instances of the data set to form successive clusters, resulting in a tree form called dendrogram. Clusters are equal to individual instances in the lowest level of the tree, and upper levels of the tree are aggregations of the nodes below. Agglomerative and divisive clustering can be distinguished, depending on whether each observation starts in its own cluster, or in the complete set.
- Partitions-based. As opposed to the previous group, partitions-based methods start from the complete data set and divide it into different disjoint subsets. Given a desired number of clusters, the process is based on assigning instances to different clusters and iteratively improving the division, until an acceptable solution is reached. Note that partitions-based methods are different from divisive hierarchical methods because, first, they require predefining the number of clusters; and second, because of their iterative nature. The well-known K-means algorithm,^135^ possibly the most commonly used clustering algorithm,^136,137^ belongs to this class.
- Density-based. If the previously described algorithms assess the similarity of instances through a distance measure, density-based algorithms rely on density measures; clusters are thus formed by groups of instances that form a high-density region within the feature space. This presents the advantage of a lower sensitivity to noise and outliers. Among the most used algorithms belonging to this family, the DBSCAN^138^ is worth mentioning.
- Probability-based. Probability-based clustering combines characteristics of both partitions-based and density-based approaches. The most important of these clustering approaches are mixture models,^139^ which are probabilistic models used to model heterogeneity and represent the presence of subpopulations (latent subgroups) in an overall population. The probabilistic component makes them a useful approach for complex (especially multimodal) data and they can be used to obtain statistical inferences about the property of latent subgroups without any a priori information about these subgroups. In practice this is achieved by using Expectation-Maximization algorithms.^140^ Important advantages are the flexibility with regards to choosing subgroup distributions and the possibility of obtaining “soft” stratification.

### Complex networks

Born at the intersection of physics, mathematics, and statistics, the theory of complex networks has proven to be a powerful tool for the analysis of complex systems. Networks are mathematical objects composed of nodes, pairwise connected by links.^141–143^ Their flexibility, and indeed their success, resides in the fact that the identity of those elements is not defined *a priori*; for instance, networks can be used to represent from people and their social connections,^144^ market stocks and their correlations or co-ownership,^145^ to genes and their co-regulation.^146^ In all cases, networks allow reducing such complex systems into simple structures of interactions, which can easily be studied by means of mathematical (algebraic) tools, while removing all unnecessary details.

The most simple way of reconstructing networks, and indeed the first one from a historical perspective, is to directly map each element composing a system to a node, and map explicit relationships between elements as links. Consider the example of a gene co-regulation network: Nodes would represent genes, with pairs of them being connected when it is known (e.g., from direct biological experiments) that one of the two genes is regulating the second. Once the full network is reconstructed, its structure can be studied through a broad set of existing topological metrics,^147^ designed to numerically quantify specific structural features; and by using these metrics as input to data-mining models.^148^

In spite of the interesting results that could be obtained through this simple understanding of networks, it was soon apparent that many real-world systems needed more detailed descriptions. Specifically, it is worth noting that a simple network reconstruction implies three hidden assumptions: that links are constant through time; that nodes are connected by just one type of relationship; and that relationships are explicit. Breaking these three hypotheses gave birth, respectively, to time-evolving, multilayer and functional networks.

### Complex systems

Systems were composed of a large number of elements, interacting in a nonlinear way between them. As opposed to more simple systems, these interactions are essential to understand the behavior of the complete system, and in some cases, they can even be more relevant than the individual elements.^149–151^ Due to this, the study of complex systems goes beyond the reductionism paradigm, where understanding is based on splitting to smaller subsystems that are simpler to understand. In other words, although the reductionistic approach works bottom-up, the systems view required to understand complex systems is a top-down one. Complex systems displays two important properties. On one hand, a nonlinear behavior, and thus tools originating in nonlinear analysis have been used in this domain—to illustrate, the analysis of time series describing the dynamics of complex systems often resort to the use of metrics of complexity,^152^ fractal dimension,^153^ sample entropy,^154^ and other types of entropies^155^ to quantify the irregularity, or detrended fluctuation analysis to quantify long-range correlations.^156^ On the other hand, emergence refers to the behaviors that may unexpectedly emerge, leading to order or disorder, and that cannot be explained by the dynamics of the system's units. Adaptation is considered as one of the qualities of complex systems, and this is a property that can be observed in the biomedical domain.^157^

### Computational drug repurposing

Drug repurposing or repositioning is the detection of novel indications for existing drugs, to treat new diseases.^158^ A major advantage of the drug repurposing strategy is that it involves approved compounds that have passed the toxicological safety screening process and have a known pharmacokinetic (PK) profile: Repositioned drugs can, hence, enter directly to clinical Phase II, making the clinical phase process much faster than newly developed drugs, and thus more cost-effective. Computational drug repurposing approaches aim at optimizing and accelerating the drug repurposing procedures, also providing means for candidate drug prioritization. Computational drug repurposing methods include the following: Structure-based virtual screening (molecular docking), Ligand-based methods (Pharmacophore model, Quantitative structure–activity relationship, and Reverse docking methods),^159^ Transcriptomic-based methods,^160^ genome-wide association study (GWAS) methods,^161^ Literature-based discovery methods,^162^ and Network-based, Multisource data integration and Machine-Learning approaches.^163^

### Constraints

In mathematics, constrains are conditions that must be fulfilled by some parameters (or solutions) of a model, to make the latter realistic. In the case of mathematical modeling of complex biological systems, different constraints can be implemented for parameters such as value range of variables, limitations of sum of parameters, transition speed, and other types of information. To illustrate, the angle of joints in the human arm cannot take any value, but must comply with some physical limitations.^164^ There are (i) general constraints that are true for any system (mass conservation, energy balance), (ii) organism level constraints—consistent limitations for all experimental and environmental conditions for a particular organism (range of viable metabolite concentrations, homeostatic constraint), and (iii) experiment-level constraints—environmental condition-dependent constraints for particular organisms (biomass composition, cellular resources).^165^

### Context awareness systems

Context awareness systems address complex environments in terms of location, identity, components, and relations. Context refers to the information that describes an entity (person, location, object).^166^ The study of such complex environments has been made possible by the availability of Wireless Sensor Networks technologies, which allow heterogeneous sensors, distributed in a physical environment, to share their measurements. Still, these technologies do not protect from problems such as cross-domain sensing and coupling of sensors; to preserve performance and reliability, the data fusion has to be performed with caution.^167^ Context awareness systems have an important role in the design of health care monitoring systems, health smart homes, and ambient assisted living, which facilitate the acquisition of both ambient and medical data from sensors. Such systems also may include reasoning capabilities consisting of data processing and analysis as well as knowledge extraction.^168^

### Correlation networks

Functional complex networks created by considering the correlation between the dynamics of pairs of nodes.

### Cross-industry standard process for data mining

CRISP-DM stands for cross-industry standard process for data mining, an industrial group that proposed a methodology for organizing the data analysis process in six standard steps.^169,170^ Since then, the term CRISP-DM has been used to indicate both the group itself and the methodology. The six steps are:

- Business (or Problem) understanding: initial understanding of the objectives and requirements of the analysis to be performed; these are expressed as a data mining problem, and should include a preliminary roadmap or execution plan.
- Data understanding: In this second phase, data are collected and a first analysis is executed, to familiarize with them; identify quality problems; discover initial insights, and formulate initial hypotheses; and identify relevant data subsets.
- Data preparation: Data received by the researchers are seldom ready to be processed; on the contrary, they usually require an initial preparation. This covers all of the activities required to construct the final data set, from selecting those data that are really relevant, to data cleaning and pre-processing. This is one of the most important steps of the whole process, as the success of the final analysis strongly depends on it; and is responsible for most of the time and resources consumed in a data analysis project, as data preparation is usually performed iteratively and without a fixed recipe. See Refs.^171–173^ for a review of techniques and the motivations for data preparation.
- Modeling: phase in which data-mining algorithms are applied and parameters are calibrated to optimal values. Some algorithms covered in this review are ANNs, decision trees (DTs), random forests (RFs), and support vector machines (SVMs). Although each one of these models has specific requirements on the format of input data, and are built on top of hypotheses on the patterns to be detected, in practice multiple algorithms are suitable in any given problem. In these situations, multiple models are optimized and compared; the models reaching a higher performance are passed to the next phase for a final evaluation.
- Evaluation: Model evaluation cannot be understood only from a data-mining perspective, for example, in terms of the achieved classification score; a business perspective should also be taken into account. Only when all relevant questions have been addressed, can one then move to the deployment of the extracted knowledge.
- Deployment: When all of the information about the business problems has been gathered, the information and knowledge then has to be organized and presented.

### Cross-validation

In data analysis, cross-validation (also known as *rotation estimation* and *out-of-sample testing*) refers to any technique used to validate a data-mining model, that is, to quantify how it will generalize to an independent data set, re-using a single data set. The initial data set is divided into multiple subsets, which are used to train or validate the model; this guarantees that the same data are never used in both tasks.^174^

### Data analysis software

With the widespread adoption of data-based solutions in many real-world scenarios, it is not surprising to find a large number of analytic solutions, spanning from cloud pipelines to commercial and freeware software, and both stemming from research activities and having a commercial nature. The most important are listed here, classified according to their underlying structure in cloud, noncloud, and hybrid tools.

#### Noncloud (or local) solutions

Commercial and freeware software tools for data analysis are designed to work on a local (or at least, noncloud) environment. In this category, one can find:

- KNIME^175^ (www.knime.com);
- SPSS Modeller^176^ (www.ibm.com/products/spss-modeler);
- RapidMiner^177^ (rapidminer.com);
- Alteryx (www.alteryx.com).

These software platforms usually have a broad focus, allowing to process any (or most) kind of data; and they allow to construct models by connecting *modules* in a graphical interface.

#### Cloud-based solutions

Also known as Platform as a Service, are solutions based on full cloud environments, and on the creation of web-based pipelines in which data are fed, processed, and returned to the user in a completely automatic way. The most notable solutions include:

- Google's ML Engine (cloud.google.com/ml-engine);
- Amazon's SageMaker (aws.amazon.com/sagemaker);
- Microsoft's Azure (studio.azureml.net).

This approach presents two advantages: a complete scalability, and a simplified user experience. At the same time, they usually provide a limited spectrum of possible analysis—for instance, Google ML Engine completely relies on Tensor Flow algorithms.^178^

#### Hybrid solutions

These solutions position themselves in between the two families previously described. Although they are designed for cloud deployment, they can easily be installed in a local infrastructure; and they shift the focus toward an intuitive representation of the results and simplified user experience. Among others, these include:

- Sisense (www.sisense.com);
- Looker (looker.com);
- Zoho Analytics (www.zoho.com/analytics);
- Tableau (www.tableau.com).

They usually allow to summarize data on high-level dashboards, with specific applications including business analytics^179^ or website usage tracking. They, nevertheless, do not provide the analytical flexibility required by systems medicine applications.

### Data fusion and data integration

Data fusion is the process of integrating multiple data sources to produce more consistent, accurate, or useful information than that provided by a single data source, whereas data integration refers to heterogeneous data obtained from different methods or sources, which are merged to produce meaningful and valuable information. In the field of system/personalized medicine, progress has been made regarding data integration, with large sets of comprehensive tools and methods (e.g., Bayesian or network-based methods), especially for multi-omics processing.^180^

### Data mining

General terms are used for describing the process of discovering patterns in data sets through the use of statistical and mathematical algorithms. Their definition overlaps with that of machine learning; and the term is also used to denote the modeling step of the CRISP-DM process.

### Decision tree

**FIG. 2.**
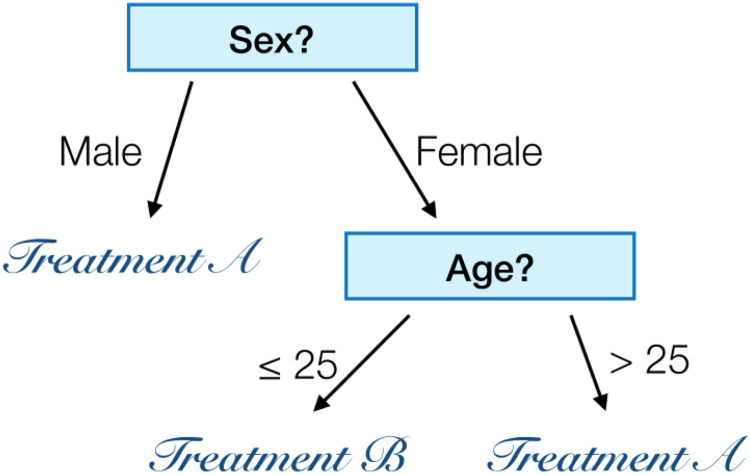
Example of a simple decision tree model, trained to choose between two treatments as a function of the age and sex of the patient.

In data mining, DTs denote classification algorithms that rely on comprehensive tree structures, and that classify records by sorting them based on attribute values. Each node in a DT represents an attribute in an instance to be classified, whereas each branch represents a value that the attribute can take—see Figure 2 for a simple graphical representation. The DTs can be generalized to target continuous values, in which case they are usually referred to as *regression trees*.

Let us denote by *D* the set of training instances that reach a node. The general procedure to build the tree is:

- If all the instances of *D* belong to the same class, then the node is a *leaf node*.
- Otherwise, use an attribute to split the set *D* into smaller subsets. These subset will then feed subsequent nodes, by applying this procedure recursively until a stop condition is met.

The main differences between the many implementations of DTs available in the literature reside in the criteria used to decide the splitting point. Among others, Gini index is used in CART,^181^ SLIQ,^182^ and SPRINT^183^; information gain is used in ID3^184^ and in the well-known C.45.^185^

The main advantage of DTs is their simplicity, both in the software implementation and in the interpretation of results; and their capacity of handling both numerical and categorical variables, thus implying little data preparation. This has fostered their use in medical applications, as reviewed, for instance, in Refs.^186,187^ They, nevertheless, suffer from a less-than-perfect performance. The concept of DT further underpins the RF classification algorithm.

### Decision support systems

Decision support systems (DSSs) are information systems, that is, systems designed to collect, process and make available information, focused on supporting different types of decisions.^188^ The DSSs typically deal with business and management challenges; can be completely customized by including multiple user interfaces and flexible architectures; and implement Optimization/Mathematical Programming tools for solution strategy and report. The DSSs are able to provide a complete view of the activities and flow within large and complex real production systems, integrating the supply of raw materials, the production phases, the products distribution, and the recovery within the sustainable and closed-loop supply chains. The DSSs in the form of standardized, enterprise-wide information systems were widely implemented in multiple sectors, including industry supply chains (e.g., pharmaceutical, manufacturing, agri-food^189^) and health care services (e.g., CDSSs^126–130^).

### Deep learning

The ANNs, which form the basis of deep learning, were developed in the 1940s as a model for the human brain.^190^ Although this model has attracted the interest of researchers in previous periods, it made a significant leap in learning and classification with the development of deep learning systems based on the layered learning structure of the human brain. One of the main reasons for this is that computational infrastructure needed to satisfactorily operate these complex structures that contain hundreds of layers and thousands of neurons have only appeared in the past decade.

**FIG. 3.**
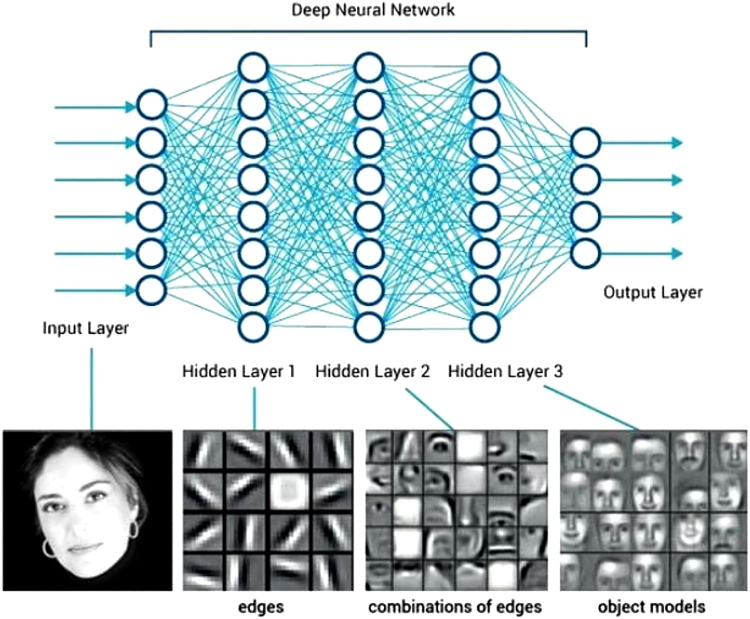
Deep-learning system developed for human face recognition. Source: https://www.quora.com/What-do-you-think-of-Deep-Learning-2

Deep-learning systems are mainly defined by the fact that each important feature of the phenomenon to be learned is automatically recognized by the algorithm and each group of features is learned by a separate artificial neural layer.^191^ For example, in an image recognition system developed for human face recognition, different facets of the face, such as lines, eyes, and mouths, and the general lines of the face are learned by different layers. Deep learning-based methods have greatly improved performance in computer vision and natural language processing, and they are integrated into many of the technologies currently used (Fig. 3).

### Digital Health

The term “Digital Health” (or d-Health) is used for denoting the massive and ubiquitous use of information and communication technologies in health, health care, and medicine fields.^192^ Digital Health covers the range of technologies used in health and medicine from genome sequencing of the microbes in the human organs, such as the gut and the skin, through genome sequencing, to the use of smartphone for supporting online telemonitoring (exposome level). The main goals of digital health are to improve health care customer follow-up and engagement, in parallel of resources and cost optimization from the health organizations and providers. As a part of the fourth digital revolution, “Digital Health” is using internet of things (IoT) and business intelligence (BI) for delivering personalized health care and medicine services. However, Digital Health is taking health care from a paternalistic medicine wherein physicians are defining and deciding how to treat the patient to being patient-centered. Patient-centered in the Digital Health context means that the electronic tools, hardware and software, are enhancing the health care customers' experience and engagement by providing them with the decision support tools for getting better health outcomes and by considering their way of life and constraints.^193,194^ Nevertheless, Digital Health reduces direct human–human interactions and thus may induce a dehumanization of health care. Within Digital Health, a subsubject has to be highlighted: the development of methods allowing improving health care customers', practitioners', and other caregivers' (like patient's family members) experience, engagement, and interactions, by considering the digital environment as another kind of point-of-care similar to clinics, pharmacies, and hospitals. One limitation of a dynamic and fast development of Digital Health lies in local regulations that have the objective of keeping health-related data and information confidential and safe, and allowing their use in ways ensuring data availability and integrity only for relevant individuals (patients and their related one when relevant, professional, and specific organizations). Digital Health is a full component of the Systems Medicine paradigm by allowing a dynamic view of individuals from the nano-level (e.g., gene expression as a response to an environmental change) to the mega-level (e.g., population interactions/reactions—discussions— on social networks as a response to an epidemic announcement).

### Digital Twin

The concept of Digital Twin is a bridge between the physical world, which can consist of a living system (i.e., an animal or a vegetal, an individual or a population) or a cyber-physical system (e.g., a biological process, a drug production line, a health monitoring service). A Digital Twin is a virtual or more accurately a computational representation of a real-world object.^195^ This kind of “duplicate” is allowing designing, implementing, and testing models in a virtual environment before or instead of performing these operations in a real-world context. From a Systems Medicine perspective, the digital twin is allowing building models of living systems (from the cell components level to the world population level for building and evaluating from biological to epidemiological models) by using socio-demographics, biological, clinical, and communicational data collected by health care customers and caregivers (see Medical Informatics section) and/or generated by IoT objects (see the Digital Health section).^196,197^

### Dissipative particle dynamics

Dissipative particle dynamics (DPD) is a stochastic simulation technique used to study dynamical and rheological properties of fluids, both simple and complex. It involves a set of particles, representing clustered molecules or fluid regions, moving in a continuous space and at discrete time steps. This meso-scale approach disregards all atomistic details that are not considered relevant to the processes addressed. Internal degrees of freedom of particles are replaced by simplified pairwise dissipative and random forces, to conserve momentum locally and ensure a correct hydrodynamic behavior.

This technique facilitates the simulation of the statics and dynamics of complex fluids and soft matter systems. The main drawback is high computing power, but this has improved due to the high performance computing, which is now combined with this technique.^198^

**FIG. 4.**
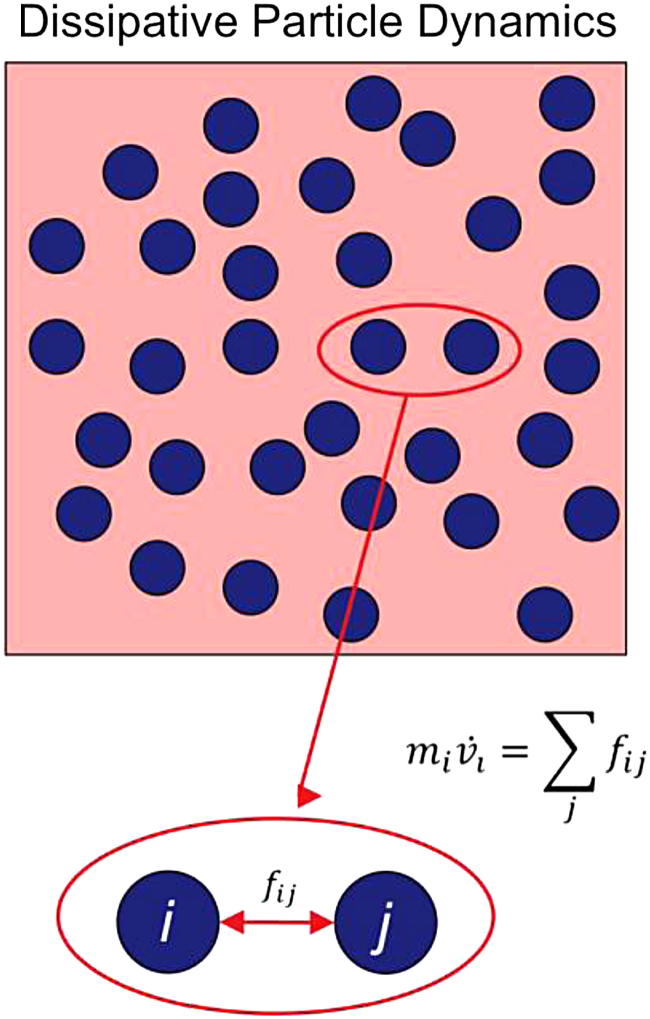
Schematic representation of a DPD model. DPD, dissipative particle dynamics.

Among others, the DPD can be used for modeling the transport of low-density lipoproteins (LDLs) through arterial wall and analyzing plaque formation, where the force of attraction of oxidase LDL molecules to the wall is modeled in the DPD solution as spring force with an experimentally determined coefficient^199^; for creating semicircular canal models with simplified geometry, showing the behavior of the fluid inside the canal, cupula deformation, and movement of otoconia particles to analyze benign paroxysmal positional vertigo^200^; or for modeling self-healing materials used for corrosion analysis and protection (Fig. 4).^201^

### Erdős–Rényi model

The Erdős–Rényi model is a model that is used to construct random graphs in which all edges, or links, have the same probability of existing, that is, they are independent. The model is usually denoted as $G\left(n,p\right)$, with *n* being the number of nodes and *p* the probability for any link to be present. Therefore, the model starts with *n* nodes, and each possible edge is included with probability *p* independent from every other edge.

The simplicity of this random network model makes it an ideal candidate for acting as null model in the normalization of network properties, although special care is required when the underlying real network is connected by construction, or has any other fixed characteristic.^202^

This simplicity also made possible the calculation of the expected characteristics of the graph, as a function of *n* and *p*, in an analytical way. Note that all these results are of a statistical nature, and hence that the error probability tends to zero; however, counterexamples can always be found. Among others, the most well-known ones include^203^:

- If np<1, then the graph will almost surely have no connected components of size larger than $O\left(\log n\right)$.
- If np=1, then the graph will almost surely have a largest component of size $\approx n^{2∕3}$.
- If $p<\frac{\left(1-\varepsilon \right)\ln n}{n}$, then the graph will be disconnected, that is, it will contain isolated nodes.
- Conversely, if $p>\frac{\left(1-\varepsilon \right)\ln n}{n}$, then the graph will likely be connected.

### Exposome

Exposome is the systems approach for disease study that takes into account the interaction of internal biological mechanisms with the environment, in other words, the interplay of genetic, epigenetic, and environmental factors. The concept was first introduced by Wild in 2005, and it encompasses for exogenous and endogenous components.^204^ A series of technological advances can be regarded as enabling technologies in this highly ambitious paradigm, including sensor networks monitor the air quality and make available the data, big data research, progress in microbiome analysis and metabolomics.

The study of endocrine disruptors and their role in pregnancy is one of the examples of this approach.^205,206^ Other work relates to cancer, and chronic diseases at large, involving pollutants, metabolism, inflammation, and diet. There are large initiatives worldwide aiming at creating synergies and building knowledge in this new field of research, as, for instance: https://www.projecthelix.eu/, https://humanexposomeproject.com/, http://metasub.org/

### Findability, Accessibility, Interoperability, and Reusability principles

In an open-science approach, making scientific research, data, and dissemination accessible, four principles for scientific data management and stewardship were defined as Findability, Accessibility, Interoperability, and Reusability (FAIR), by the Force11 working group (https://www.force11.org/^207^). The principles do apply not only to data but also to algorithms, tools, and workflows. These objectives are now becoming expectations from funding agencies and publishers, regarding the use of contemporary data resources, tools, vocabularies, and infrastructures to assist research discovery and reuse by third parties.

### Feature selection

In data analysis, the process of feature selection consists of applying algorithms designed to select a subset of features, from the original data set, for subsequent analysis. All other features are ideally irrelevant for the problem at hand, and they are thus disregarded.

Feature selection yields two main benefits. On one hand, even when the studied data set is not of a large size, it can help in data understanding, reducing training times, and improving prediction performance. On the other hand, feature selection is essential when the features outnumber the instances. To illustrate, domains such as gene and protein expression, chemistry or text classification are characterized by the limited availability of instances to train models—for example, a few patients and control subjects, a few complete textual records, etc. Refs.^208,209^ extensively review methods for feature selection.

Feature selection methods are usually classified in three different families:

- Filters select subsets of variables, according to some rules, as a preprocessing step; in other words, this selection is not made taking into account the subsequent classification. One of the most relevant examples is the recursive feature elimination, based on iteratively constructing a classification model and removing features with low weights (i.e., of low relevance)—note that the classification model used here is independent from any subsequent classification. When features are added, instead of being eliminated, the result is a forward strategy.
- Wrappers assess subsets of features according to their usefulness to the subsequent classification problem. When the number of variables is reduced, this is done by evaluating all possible variable combinations; on the other hand, when this is not computationally feasible, a search heuristic is implemented. Note that here the machine-learning algorithm is taken as a black box, that is, it is only used to evaluate the features' predictive power. Wrappers can be computationally expensive and have a risk of overfitting in the model,^210^ in which case coarse search strategies may be applied.
- Embedded techniques are similar to wrappers, but they integrate the search of the best subset of features within the classification model.^211^ The classification is then formalized as an optimization of a two-part objective function, with a goodness-of-fit term and a penalty for a large number of variables. Embedded methods that incorporate variable selection as part of the training process may be more efficient in several aspects, as they make better use of the available data and are more computationally efficient. On the negative side, they are specific to a single learning algorithm, and are thus not generalizable.

### Finite element method

Finite element method (FEM) is a numerical method that is used for solving problems in different fields of engineering and mathematical physics. They can be widely categorized into structural analysis, heat transfer, fluid flow, mass transport, and electromagnetic potential. The FEM formulation of the problem requires solving a system of algebraic equations. Analytical solutions of these problems generally require the solution to boundary value problems for partial differential equations. The domain of interest is divided into a finite number of simpler parts called elements, and the method calculates values of the unknowns at discrete number of points over the mentioned domain. The simple equations at each point of the model are then assembled into a larger system of equations that describe the entire problem. Analysis that is associated with solving a problem using FEM is called finite element analysis.^212,213^

**FIG. 5.**
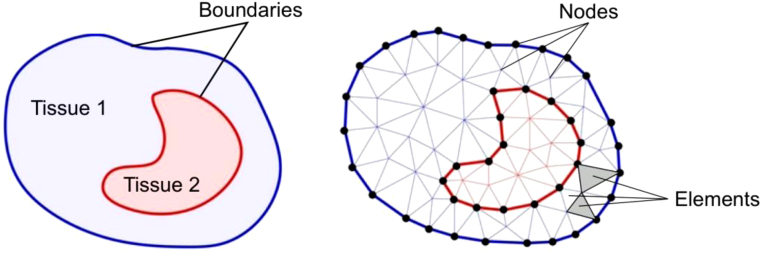
Schematic representation of an FEM model. FEM, finite element method.

Examples of the application of FEM in medicine include the analysis of bone—hip implant interactions, to obtain the information about shear stress distribution^214^; the development of several inner and middle ear models, especially cochlea models and their analysis^215^; the computational model of arteries^216–218^; the detection and localization of ischemic cardiac diseases^219^; or the examination of electrospinning jet trajectories (Fig. 5).^220^

### Finite volume method

**FIG. 6.**
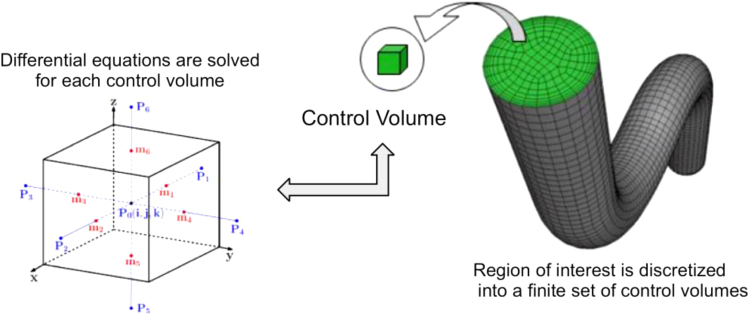
Schematic representation of an FVM model. FVM, finite volume method.

Finite volume method (FVM) is a method that uses an approach to represent and solve partial differential equations in the form of algebraic equations. The term “finite volume” marks a small volume that surrounds each point (called node) in a mesh. By dividing the domain of interest in the form of mesh (structured or unstructured mesh), this method leads to robust schemes. Different conservation laws are used—elliptic, parabolic, hyperbolic, etc. The FVM is often chosen when flux is of interest, since local conservativity of the numerical fluxes (conserved from one discretization cell to its neighbour) is a characteristic of this method. This is especially present in the field of fluid mechanics, semi-conductor device simulation, heat and mass transfer, etc. By local conservativity it is meant that an integral formulation of the fluxes over the boundary of the control volume is obtained. A local balance is written on each discretization cell, which is called “control volume.” The fluxes on the boundary are discretized with respect to the discrete unknowns.^221^ The FVM can, for instance, be used in PK models (Fig. 6).^222^

### Frequentist statistics

Frequentist statistics is an interpretation of statistics that considers the probability of a random event as being the long-run (in the sense of Neyman, Pearson and Wald tradition) proportion of occasions on which it occurs, conditional on some specified hypothesis.^68^ For a different interpretation, see the Bayesian Statistics section.

### Functional networks

In all original studies focusing on complex networks, one inherent hypothesis was the fact that the structure of the network was easily observable: For instance, neural connections in the *Caenorhabditis elegans* can be obtained by physically looking at the organism. However, many real-world systems do not comply with this requirement: Their structure is not observable, and we can only measure some aspects of the dynamics of the constituting elements. If one makes the hypothesis that the dynamics of each element is partly the result (or “the function”) of the dynamics of its peers, then the structure of interactions can, in principle, be inferred from the individual dynamics: The result is called a functional network. The introduction of this latter representation has resulted in an important step forward in network science, allowing a broader focus including both structural and dynamical (functional) relations, and shifting the focus from the underlying physical structures to the flow of information developing on top of them.^223,224^ Although a detailed description of the functional network theory is beyond the scope of this review, it is worth reporting a sketch of the standard way of reconstructing them. Let us suppose that a set of time series is available, each one describing the dynamics of one element (node) of the system; to illustrate, in neuroscience these typically correspond to measurements of electric (EEG) or magnetic (MEG) fields generated by the brain, or the consumption of oxygen by neurons (functional MRI). The synchronicity between the dynamics of pairs of nodes is then estimated, using metrics such as linear correlations or causalities. Finally, the resulting functional networks can be analyzed alone, that is, as standard networks^148^; or the relationships between the physical substrate and the functional connectivities can be explored.

### Gene set enrichment analysis

The methods to identify sets of functionally related genes are enriched or depleted when comparing two biological states.^225^ It does not require that individual genes are statistically scored as significantly altered, as it ranks all genes and compares this rank list with predefined sets of genes, usually designated as molecular signatures. Since it does not require any definition of a threshold for up- or downregulation, it can identify even weaker changes of gene expression, which are significant for a gene set, but not for a single gene. The gene sets or molecular signatures used for the comparison with the rank list are accessible through a public repository, and they are based on known biological functions, pathways, or cell types.^226,227^ Computation of the gene set enrichment can be performed with open software or a web platform of the Broad Institute (http://software.broadinstitute.org/gsea/index.jsp)^226^; on other web sites such as *Enrichr* (http://amp.pharm.mssm.edu/Enrichr/), or with packages of the Bioconductor R environment (https://www.bioconductor.org/). Other tools can also be used within the gene set enrichment analysis (GSEA) software:

- Leading Edge Analysis: Examines the genes that are in the leading-edge subsets of the enriched gene sets. A gene present in many leading-edge subsets is likely to be of interest.
- Enrichment Map Visualization: Cytoscape plugin for functional enrichment visualization (www.baderlab.org/Software/EnrichmentMap)
- Chip2Chip: Converts the genes in a gene set from HUGO gene symbols to the probe identifiers for a selected target chip.
- GSEAPreranked: Runs the GSEA against a ranked list of genes, which you supply (e.g., mRNAseq).
- CollapseDataset: Creates a new dataset by collapsing each probe set into a single vector for the gene, which is identified by its HUGO gene symbol.

He GSEA can also be improved by integrating external information, for example, pathway or ontology information; some of the previously described software packages, including *Enrichr* and the *Bioconductor R* environment, include functions to perform this analysis.

### Granger causality

Granger causality is a statistical method allowing to infer cause–effect relationship between events, or corresponding variables, through exploitation of the concepts of explained variance and prediction. According to Granger,^228^ a signal X “Granger causes” Y if current and future values of Y can be better predicted using current and past observed values of X. Although formally known as Granger causality, this statistical method can be seen as a practical application of the earlier research in causality.^229^ Since its formulation in the late 1960s, Granger causality has been widely used in economics. As a result, Prof. C.W. Granger received the Nobel Prize in Economics in 2003.

The Granger causality has extensively been used in neuroscience, and specifically for the reconstruction of functional networks representing brain dynamics^230,231^ and of physiological networks in general.^232^ More in general, this metric allows describing the causal relationship between pair of time series; it has thus been used to assess aspects from cardio-respiratory instability events,^233^ to the relationship between health care expenditure and its output.^234^

### Graph embedding

Graph embedding (also known as network embedding) is a representation of a graph in a vector space, where relevant graph features are preserved. Their advantage resides in the fact that vectors are easier to handle than full graphs in several domains of machine learning.^148^ A lot of graph embeddings methods have been proposed for graph analysis in the following areas: nodes classification, edges (link) prediction, clustering, and visualization. Graph embedding methods are categorized into three broad categories: (i) matrix factorization based, (ii) random walk based, and (iii) neural networks (or deep learning) based.^235^

There are several challenges that need to be considered for using graph embeddings. The biggest challenge in learning a graph embedding is the choice of metrics, node and edges properties, and features to be preserved in the vector representation. The learnt embeddings should represent the rich graph information, including topological structure and auxiliary information. Moreover, the graph has to be constructed in a way to represent nodes relations as well as to maintain the node proximity matrix in embedded space.^236^ Next, different application domains have different prerequisites for a using a suitable graph embedding algorithm. Therefore, the embedding dimensionality decision based on graph size should meet application requirements. Unfortunately, it has been argued that in several real-world complex network applications, graph embeddings cannot represent the network's most important features.^237^

In the biomedical domain, graph embedding methods can be used to represent graphs for PPIs,^238^ brain regions connections,^239^ infectious diseases modeling,^240^ chemical reactions between metabolism enzymes,^241^ or regulatory genes interactions.^242,243^ These give an overview and comparison of the use of graph embedding methods in three important biomedical link prediction tasks: drug-disease association prediction, drug–drug interaction prediction, and protein–protein interaction prediction; and two node classification tasks: medical term semantic type classification and protein function prediction.^244^ These identify relevant gene functions for a biological context using network representation learning with a neural networks-based graph embedding method. In a neuroscience context, a random walk-based graph embedding method is used for embedded vector representations of connectomes to map higher-order relations between brain structure and function.^245^

### Hidden conditional random fields

Hidden conditional random fields (HCRFs) are discriminative latent variable models, used for the classification of sequences of events; in other words, these models are useful to process inputs that are graphs of local observations.^246^ Given one sequence, the HCRF tries to assign a single label to it, by introducing a set of latent variables corresponding to each element of the sequence, and by conditioning the label to those variables. Beyond providing rules to discriminate one label from all the others, HCRFs also yield the structure that is shared among labels. This classification model has been proved to be efficient, provided enough instances are available to validate the hidden structure. Although still not widespread in the medical domain, some applications of HCRFs include the analysis of brain dynamics^247^ or the recognition of protein folding structures.^248^ The main limitation of HCRFs is that no rules are presently known to define the optimal number of hidden states for a given problem; the solution, that is, a trial-and-error process with cross-validation, can be computationally expensive.

### Imputation

In statistics and data analysis, imputation refers to the set of techniques and algorithms used to handle missing data in the raw data set. These can be divided into three categories:

- Listwise deletion, that is, the strategy of deleting any instance containing missing data. This approach, though extremely simple and easy to implement, can only be used when data are missing at random (as otherwise the deletion would introduce a bias), and when a large number of instances is initially available.
- Single imputation. Missing values are substituted by new values, according to some rules, and a new data set is therefore created. Techniques include hot-decking (when instances with missing values are substituted by other instances, chosen at random) and mean or median substitution (the missing value is filled with the mean or median of that feature).
- Multiple imputation. Missing values are replaced by values generated according to a statistical rule, for example, Multiple Imputation by Chained Equations^249^ or Latent Class Analysis.^250^ Multiple imputed data sets are generated and are analyzed in parallel, for then extracting a single consolidated result.

Imputation is never perfect nor without impact. The choice of optimal missing value treatment depends on multiple factors, including the nature of data and their correlations, the amount and randomness of missing values.

### *In silico* modeling

*In silico* modeling involves the development of computer models to simulate a pharmacological or physiologic process.^251–254^ It is an extension of controlled *in vitro* experimentation. Although mathematical electrophysiological models exist for decades (e.g., in electrophysiology of the heart), the increase in computing power available for research purposes with lower price has enabled larger scale models, for example including the cell nodes for a whole heart and incorporating personalised organ geometry based on medical imaging. Specialized platforms allow for executing the simulations and solving the numerical problems, nowadays typically in high-performance computing infrastructures. *In silico* modeling combines the advantages of both *in vivo* and *in vitro* experimentation, with the main advantage of not being subjected to the ethical considerations and lack of control that is the case with *in vivo* experiments. *In silico* models theoretically allow unlimited array of parameters to be included, contrary to the *in vitro* experiments that exist in isolation. This means that the results would be more realistic and applicable to the organism. The PK experimentation is often connected to the *in silico* modeling. In addition, complex *in silico* models have been applied to pathophysiological problems to provide information that cannot be obtained practically or ethically by traditional clinical research methods. These models have enabled to obtain valuable information in many fields—pure physiology, congenital heart surgery, obstetric anesthesia airway management, mechanical ventilation, and cardiopulmonary bypass/ventricular support devices. In spite of many advantages, the interested researcher should also be aware of one main drawback of *in silico* modeling, that is, that not all strategies have been validated *in vivo*.^255^

### Integrative analysis

“Integration” may have different connotations, depending on the context.^256^ In its most general sense, it refers to combining things, such as two viewpoints, or multiple systems, or multiple data sets. For life science data and in particular functional genomics, Lu et al.^257^ defined data integration as the “process of statistically combining data from different sources to provide a unified view and make large-scale statistical inference.” For multi-omics data integration, clearly this definition is too limited, in that it only refers to statistics as a means and underappreciates the opportunities that lie in creatively combining analytic methodologies (for instance, statistics and machine learning). A more challenging definition for data integration in complex disease analysis involves the process of combining data within a generic framework that encompasses organizing principles for the interaction of different types of systems. This definition does not explicitly refer to statistical, bioinformatics, or computational tools but to any approach that fits within a transdisciplinary viewpoint. It includes data fusion as well as more fancy and more elaborate forms of combining evidence from different data sets or sources.^258^ Further, it agrees with the definition of Thorsen and Oxley^259^ as the process of connecting systems (which may have fusion in them) into a larger system. Apart from data integrative analysis, integrative analysis sometimes also refers to the integration of analytic tools or methods, to combine different analytic viewpoints to the same data.

### Interactome

A map represents the whole set of molecular interactions in a particular cell. Although usually interactome specifically refers to physical interactions, it can also be used to describe sets of indirect interactions among genes. As molecular interactions can occur between any pairs of molecules composing the cells (including proteins, nucleic acids, lipids, carbohydrates, and so forth), a great number of interactome maps can be defined; nevertheless, the most common and well known include:

- The PPI and (PIN);
- The protein–DNA interactome, also called a GRN, a network formed by TFs, chromatin regulatory proteins, and their target genes;
- Metabolic networks, representing metabolites and how they are converted into each other by enzymes.

For the corresponding mathematical representations of such maps, see the Biological Networks section.

### Internet of things

IoT is related to the evolution of the internet toward integrating real, everyday life devices called things.

A comprehensive description is provided in Vermesan et al.^260^: IoT “is a concept and a paradigm that considers pervasive presence in the environment of a variety of things/objects that are able to interact with each other and cooperate with other things/objects to create new applications/services and reach common goals.” Thus, IoT aims at achieving a virtual representation of a set of physical devices through the deployment of technologies and architectures involving large-scale, loosely coupled systems.

Generally speaking, basic IoT systems components include: IoT Standards and Ecosystems, Event Stream Processing, IoT Device Management, IoT Platforms, IoT Analytics, and IoT Security.^261^ An important aspect is the IoT Reference Model, the model that defines all architectural aspects of the system, and which is composed of the following sub-models: IoT Domain Model, IoT Information Model, IoT Functional Model, IoT Communication Model, and IoT Security Model.^260^ Moving from a theoretical to a physical representation of IoT, this is usually composed of: Smart devices, Network, Data processing, Data storage, Data aggregation, data analytics, and process integration.

Communication between IoT elements can be addressed through multiple paradigms: device-to-device communication, device-to-IoT platform communication, device to gateway, and data aggregation. The relationship between IoT and multiscale computing and multiscale modeling and simulation can be related to the following components: IoT as data provider for Multiscale Modeling and Multiscale Modeling as a way to experiment and validate complex processes with the aid of IoT.

Many synergies have been found between IoT systems and Multiscale Modeling. First of all, IoT can facilitate data provision to the modeling phase, by handling access, routing, and recording of data acquired from sensors attached to smart objects. Second, IoT devices naturally measure the physical space at different resolution and conceptual levels, thus providing a multiscale view of the space. In addition, IoT can simplify the understanding of the raw data through technologies related to Big Data, semantic representations, ontologies, and machine-interpretable representations of domain knowledge, and context awareness.

Multiscale IoT Systems for Experimental Multiscale Models can be used to acquire data at multiple scales corresponding to the scales selected in the Multiscale Model. Such IoT systems design use multiscale principles. The complex processes include Machine-to-Machine and Human-to-Machine Interaction. Relevant enabling technologies are related to Heterogenous objects, Heterogenous distributed systems (P2P, Wireless Sensor Networks, Cloud Computing), and Complex Systems of Systems. The IoT as a complex systems is not a simple set of subsystems and involves data and energy transformation, interaction, interoperability, feed-back and feed-forward structures, self-organization, and self-management.^262^

An important development of IoT with applications in medicine is referred to as internet of medical things (IoMT). The IoMT can be described as an internet-based environment connecting medical devices and services. Applications of IoT technologies in medicine are increasingly common.^263–265^ In cancer treatment studies, blood pressure monitoring bracelets and tracking apps have been used to gather relevant information. Continuous glucose monitor can be connected in an IoT environment to transmit data to mobile devices, thus facilitating the analysis of blood glucose levels. A Bluetooth-enabled coagulation system has been used in connection to the IoT environment to help patients become aware of potential blood clots and transmit results to health care providers. A wearable smart asthma monitor can detect symptoms related to asthma attacks and connected to an IoT environment it can track and detect the inhaler.

### Lattice Boltzmann method

Lattice Boltzmann (LB) method is a discrete numerical method used mainly for simulations of fluid flow.^266–270^ The main advantage of this method is that it is not necessary to solve differential equations, which makes the implementation relatively simple and it is possible to parallelize the software. In the LB method, fluid is observed as a set of fictional particles. These particles can move along the predefined directions, and the dynamics of their motion is modeled through their mutual collisions and further propagation in the observed domain. A special distribution function is defined, and this function depends on the state of neighboring particles and has an identical form for all the particles, that is, for all the nodes in the lattice mesh. Macroscopic quantities, such as density, pressure, and velocity, are calculated by using the components of the distribution function.^271,272^

**FIG. 7.**
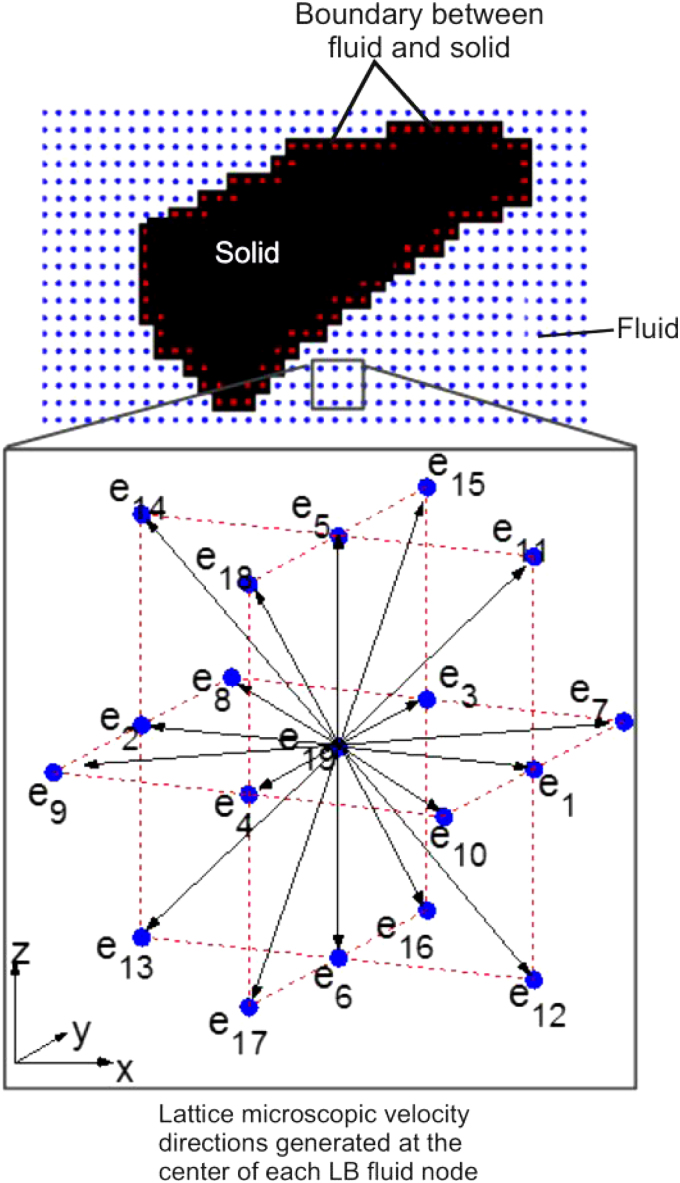
Graphical representation of the LB method. LB, Lattice Boltzmann.

Examples of the use of the LB method in medicine include the modeling of the motion of endolymph through the semicircular canals of the inner ear^273,274^; and the analysis of the numerical and experimental transport of LDLs through arterial walls.^199^ Open-source software implementing LB methods are also available; see, for instance, https://www.openlb.net and https://palabos.unige.ch (Fig. 7).

### Machine learning

Machine learning is the science of using computers to discover new information from observations.^275,276^ There are several families of machine-learning methods: supervised learning, unsupervised learning, and semi-supervised learning. The choice of the strategy depends on the nature of the used data. A large and complex database is commonly required to develop a machine-learning model. In the system medicine field, bio-marker extraction or human genome classification is a typical example of a machine-learning model. For further details, see also data mining, CRISP-DM, deep learning.

### Mediation analysis

If two variables (an independent *x* and a dependent *y*) show a statistically significant correlation, it does not necessarily mean a direct causative link, as the correlation might be caused by a third variable (the mediator), which is often nonobservable—and which is influenced by the independent variable and by itself influencing the dependent variable. A mediation analysis can elucidate such interactions and dependencies and it helps to differentiate between direct and indirect effects.^277,278^ This type of analysis can be performed with specific packages of the Bioconductor R environment or with add-ins of commercial software such as SPSS. It is important to note that a mediation effect can be full or partial—and that it can be moderated by additional parameters. In addition, it has to be stated though that mediation analysis cannot be used to detect or analyse multiple interdepencies.

### Medical informatics

Medical informatics (also known as Health Informatics or Biomedical Informatics) is a science at the crossroad of information science, computer science, social sciences, and health and medical sciences. This research area deals with all the components of information systems (data acquisition, information and knowledge resources, devices and networks, regulation and ethics, and more) used for supporting and improving health care management (e.g., clinical knowledge management), delivery (e.g., patient-related data follow-up over time), and research (e.g., developing standards encoding diagnostic for epidemiological purposes).^279–282^ Medical Informatics is an umbrella and the core for different sub-specialities such as clinical informatics, nursing informatics, public health informatics, consumer health informatics, and veterinary informatics. As a multidisciplinary field, the Medical Informatics playground consists of developing and investigating theories, models, methods, processes, and systems, used for generating, storing, retrieving, using, and sharing health and medical data, information, knowledge, and decision support. From an application perspective, medical informatics is actively and dynamically investigating and supporting health and medical reasoning by experimenting models and simulations across a wide spectrum: from molecules to populations, from a biological system point-of-view to a global population and One Health perspective. Moreover, end-users are a crucial component of the overall system in Medical Informatics. For efficiency reasons, researchers in the field of Medical Informatics have to continuously monitor the changes in different spheres such as the social, economic, ethical, and educational, and update their models in accordance to these changes. In recent years, there has been an important and growing trend of applying algorithms and know-how from the fields of BI and automation in Medical Informatics, for example, data and text mining, analysis, and information and knowledge management—see the Clinical Decision Support Systems section. From the integrative perspective of systems medicine, Medical Informatics investigates and delivers end-to-end frameworks supporting complex medical decisions, driven by evidence-based medicine for continuously improving health and disease management at the individual and populations levels.^283^ One of the most critical parts of research done in Medical Informatics considers ethical and legal regulations and constraints in the technological side of medical field.^284^ As new means of measuring, communicating, and managing patients emerge, there is a need to continuously monitor and update the requirements for ensuring security, that is, keeping confidentiality, integrity, and availability of health and medical-sensitive data.

### metaboAnalyst

Part of the same family of websites including networkAnalyst and microbiomeAnalyst, this website provides a visual analytics platform for meta-analysis of metabolomics data (www.metaboanalyst.ca).^285^

### Metabolomics

Metabolomics is the scientific study of a set of metabolites present within an organism, cell, or tissue. It was also defined as a global measurement of small molecules (metabolites), which are produced or modified in an organism. Metabolites can also result from a stimuli (nutritional intervention, drugs, genetic perturbations, etc.), are present in a system (blood, urine, saliva, etc.), and are accessible to analysis.^286,287^ Metabolomics is one of the functional level tools being employed to investigate not only the complex interactions between metabolites but also their regulatory roles through their interactions with genes, transcripts, and proteins. It is actually considered as a powerful phenotyping tool to better understand the biological mechanisms involved in the pathophysiological processes and identify biomarkers of metabolic deviations.^288^ Indeed, it provides, at a molecular level, multivariate information of multi-compartmental biological systems that reflect changes in biological processes.^289^

### microbiomeAnalyst

Part of the same family of websites including networkAnalyst and metaboAnalyst, this website provides a visual analytics platform for meta-analysis of microbiome data (www.microbiomeanalyst.ca).^290^

### Model robustness

Model robustness is a widely used concept in modeling under uncertainty, namely with Robust Optimization approaches. For that, the objective function of a Stochastic Linear/Quadratic Programming is modified by introducing penalization parameters related with nondesired attributes (e.g., high variability on solutions, nonsatisfaction of products demands, over-designing of production capacities, nonutilization of expensive equipment), or probabilistic restrictions are modified by enlarging/narrowing “soft” bounds (e.g., “worst case” analysis).^291^

For instance, the Two-Stage Stochastic Programming (2SSP)^292^ approach for the capacity expansion of a pharmaceutical supply chain allows both the promotion of solution robustness (by penalizing the deviations on the solutions, e.g., minimizing the solutions variance) and the model robustness (e.g., minimizing the expectances for the nondesired attributes). Namely: (i) at the first stage, the capital and investment decisions must be taken (i.e., the project variables are calculated “here-and-now”); (ii) in the second stage, the uncertainty is introduced through a set of scenarios and the related probabilities (in this “recourse phase,” it occurs through the probabilistic calculation of the control variables).

Then, model robustness is obtained when the optimal solution does not present high values for the probabilistic measures of the attributes to avoid (namely: for the expectance of excess/unused production capacities that would imply larger investment costs; and for the expectance of unsatisfied products demands that would impact negatively the patient's health). Model robustness is also strongly connected with other concepts of interest, such as Model Verification and Validation, Parameter Sensitivity Analysis and Uncertainty Quantification, and Probabilistic Risk Analysis (PRA). Several drawbacks can occur on model robustness developments, for example, due to resource consuming, standard accuracy, or uncertainty; see Refs.^293,294^ for details.

### Model verification and validation

Model verification is a process to verify whether a given model has been directly coded or mathematically represented; on the other hand, model validation aims at verifying whether the implemented model is the right one for the biological system of interest. Model verification is a straightforward task, thanks to many direct techniques to check and debug computer programs. Model validation, on the other hand, is more complex, and is commonly performed by using theoretical outcomes or experimental measurements. It is important to note that model validation of biological systems is extremely complex and difficult due to the lack of *in vivo* data and measurement protocols.^295,296^

### Morphometric similarity networks

Morphometric similarity networks (MSNs) are graph-based representations of the structure of the brain.^297^ The study of structural differences in the brain by topological analysis based on graph theory has the disadvantage of generating a connectivity matrix at the group level and, therefore, the connectivity parameters are calculated at the group level. Recently, a new technique has been developed that allows to generate a connectivity matrix at subject level based on the interregional similarity of multiple morphometric parameters measured by multimodal MRI.^297^ Typical morphometric measurements taken from multimodal image data for each brain region are: fractional anisotropy, mean diffusivity, magnetization transfer, gray matter volume, surface area, cortical thickness (CT), intrinsic (Gaussian) curvature, mean curvature, curved index, and folding index. For each subject, these values will form a vector of morphometric measurements for each region. Then, the morphometric similarity matrix (MSM) of the subject will be obtained by calculating the Pearson's correlation between the vectors of the morphometric characteristics of each pair of regions. Finally, the MSN will be obtained by thresholding this MSM. Therefore, we end up with one network (MSN) per subject, which will allow us to calculate the (structural) connectivity parameters at the subject level. Recently, some papers have been published that demonstrate the validity of this technique.^298,299^

### Multiphysics systems

Multiphysics systems are systems consisting of more than one component, each governed by its own principle(s) for evolution or equilibrium (conservation or constitutive laws).^300^ Two possibilities for classification are related to the coupling:

- bulk couplings, that is, through relations that are active in the overlapping domains of the individual components;
- couplings happening on idealized interfaces of lower dimension, for example, through boundary conditions that transmit fluxes, pressures, or displacements.

Some examples of bulk-coupled multiphysics systems include radiation with hydrodynamics in astrophysics, electricity and magnetism with hydrodynamics in plasma physics (magnetohydrodynamics), and chemical reaction with transport in combustion or subsurface flows (reactive transport). Since forward models are simulated successfully, inverse problems, sensitivity analysis, uncertainty quantification, model-constrained optimization, and reduced-order modeling are gaining more attention. The physical model is, in these advances, augmented by variables other than the primitive quantities in which the governing equations are defined. These variables may be sensitivity gradients, probability density functions, Lagrange multipliers, or coefficients of system-adaptive bases. Equations that govern the evolution of these auxiliary-dependent variables are often derived and solved together with other physical variables.^301^ For an example of applications of multi-physics systems to medicine, see Šušteršič et al.^220^

### Multilayer networks

**FIG. 8.**
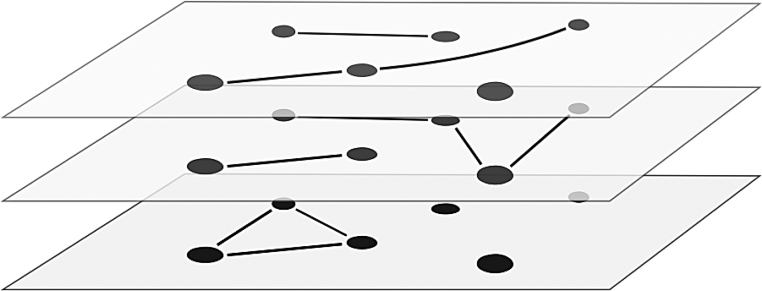
Example of a graphical representation of a multilayer network composed of three layers.

Complex networks are interactions that are defined on more than one layer. In the standard complex network approach, links between nodes are usually of a single type, the only difference between them being a (generally, real) number, quantifying the weight of the connection. Nevertheless, considering all links as homogeneous can be an important constraint, as connections in real-world systems may be of different types. A biological example can help clarify this. One of the most interesting kinds of success in recent neuroscience has been the creation of a full map of the *C. elegans*' neural network, consisting of 281 neurons and around 2000 connections.^302^ However, connections are not homogeneous: Neurons can communicate through chemical and electrical (ionic) links, with completely different dynamics and time scales. Therefore, a correct representation should include two independent layers of connections. This resulted in the creation of the multilayer network concept, that is, graphs whose connections are organized in separate layers.^303^ Multilayer networks explicitly incorporate such heterogeneity, such that each link type (relationship, activity, category) is represented by a different layer, with the same node having different neighbors in each layer (Fig. 8).

### Multiscale biomolecular simulations

Biomolecular simulations are computer simulations of molecular dynamics of biological systems, such as proteins, nucleic acids, saccharides, membranes, and their complexes. Multiscale biomolecular simulations are simulations of molecular dynamics of biological systems at different levels of granularity, differing in spatial resolution and other aspects.

The first attempts to simulate molecular systems started in 1950s. The first biomolecular simulation was published in 1977 by McCammon et al. (2013 chemistry Nobel Prize winner).^304^ The authors simulated several picoseconds of bovine pancreatic trypsin inhibitor in vacuum. An important milestone of biomolecular simulations was the development and refinement of biomolecular force fields (formulas and their parameters for calculation of potential energy from atomic coordinates) and simulation software. Packages CHARMM, AMBER, Gromos, Gromacs, NAMD, ACEMD, and BOSS have been tuned for high performance on a wide range of machines and operation systems.

There are several types of granularity in multiscale biomolecular simulations. The main reason for interest in multiscale versions of biomolecular simulations is in the fact that these simulations are extremely computationally expensive. Each atom in a typical solvated biomolecular system interacts (covalently or noncovalently) with another ∼5000 atoms. These interactions must be evaluated in every simulation step. The integration step of most biomolecular simulations is in a femtosecond scale. It is, therefore, necessary to carry out millions of steps (and evaluate interactions of millions of atomic pairs in each step) to simulate nanosecond time scales.

The first type of granularity is in the modeling of interaction between atoms. There are two major models that make it possible to calculate energy and forces in a molecular system—quantum mechanics and molecular mechanics. Quantum mechanics models the system by solving Schrödinger equation for electrons. On the other hand, molecular mechanics represents atoms as particles connected by simple mechanical “springs” and interacting via interatomic potentials with simple mathematical descriptions. Electrons are not explicitly modeled. Quantum mechanics calculations are significantly more complex and, therefore, more computationally expensive. The advantage of quantum mechanics is that it does not require *ad hoc* sets of parameters for each class of molecules. Further, most molecular mechanics models do not take into account the reactivity of the molecular systems. Molecular mechanics (with a few exceptions) keeps the chemical structure fixed during the whole simulation, that is, it disallows breakage and formation of covalent bonds in chemical reactions. For this reason, quantum mechanics is used to study the mechanism of chemical reactions.

Enormous computational costs of quantum mechanics led to a mixed (multiscale) model of quantum mechanical and molecular mechanical (QM/MM) calculations. For example, an enzymatic reaction can be studied on a model of enzyme with the substrates and active-site residues modeled by quantum mechanics and the rest of the system modeled by molecular mechanics.

This second type of granularity addresses the number of particles in the molecular system. These models differ in the number of atoms represented by a single particle. In a standard fine-grained (“all-atom model”) model, there is one particle representing one atom. All quantum mechanical models are all-atom models. Simplified versions called “united-atom models” represent certain groups of atoms, such as CH, CH_2_, and CH_3_, as a single particle. Such a particle represents the bulk properties of the whole group. This reduces the overall number of particles in the system and accelerates the simulation without significant loss of resolution.

Further coarse-graining in so-called “coarse-grained models” replaces multiple atoms, typically four nonhydrogen atoms, by a single particle. Coarse-grained simulations make it possible to study several orders of magnitude longer time-scales than all-atom simulations. The prize paid for this is loss of resolution. Coarse-grained simulations have been extremely successful in simulations of membranes, interfaces, and related systems. They are less frequently used in studies requiring precise atomic resolution, such as in drug discovery. Models mixing all-atom and coarse-grained simulations (similarly to mixed QM/MM models) have been developed to address this problem.

There are examples of studies with further coarse-graining. For example, elastic network models of proteins represent individual amino acids as particles connected by harmonic springs. This representation of a protein resembles models used in civil engineering to test mechanical stability of constructions. They are used in biomolecular simulations, but more frequently, they are studied by static approaches such as normal mode analysis. Surprisingly, bulk mechanical properties of biomolecules can relatively be accurately predicted by using such simplified models.

The major aim of biomolecular simulations is to predict a certain property of the biomolecular system. The third type of granularity is in the depiction of such molecular properties. Biomolecular simulations produce trajectories—thousands of snapshots of thousands of atoms. These pieces of big data can be analyzed to extract relevant low-dimensional properties of the systems. Such properties can be then used to build thermodynamic and kinetical models of the simulated system.

The last granularity is the computational granularity. As already mentioned, biomolecular simulations are computationally expensive. Most software used in biomolecular simulations has been developed to run in parallel on multiple cores of a CPU (multithreading) and multiple CPUs and nodes connected by Message Passing Interface. Recently, Fast Multipole Method^305^ is being introduced into biomolecular simulations to enable multiple levels of parallelism. Alternative hardware such as graphical processing units and special purpose hardware have been successfully used. The multiscale nature can be further extended by application of special multiple ensemble or multiple time-scale methods.

### Multiscale modeling

Multiscale modeling is a numerical approach that is used to study the biological systems of interest at multiple time and length scales, that is, in which multiple models at different scales of time and/or space are used simultaneously to describe one complex system.^306^ To illustrate, a multicellular organism can be modeled at different levels, for example, DNA, cells, fibers, and tissues; with each model getting input from the lower-level one.^307^

Those models are commonly developed by using a combination of several numerical methods. The FEM could be used to model system behavior at organ and tissue scales. Agent-based simulation could be used to model single-cell or cell population behaviors. Molecular dynamics could be used to describe the movements of atoms and molecules. To make the link between scales, the homogenization theory could be used. This theory allows constitutive behaviors at the macroscopic level to be described by using the information from interactions between macroscopic and microscopic levels. There are two main multiscale modeling strategies. The first one is the hierarchical simulation, in which the system behavior is separately described and simulated for each scale and then the interaction is performed. The second one is the concurrent simulation, in which all system behaviors and their interactions are simultaneously described and simulated. There is no time delay by using the second strategy, but the strategy is complex for model development and implementation.

**FIG. 9.**
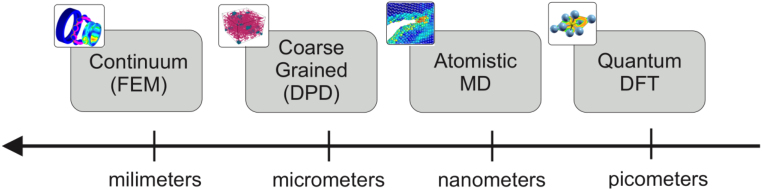
Graphical representation of the typical scales in a multiscale modeling.

The importance of multiscale modeling lies, on one hand, in the fact that available macroscale models are usually not accurate enough, and on the other hand, in the fact that microscale models are not efficient enough and/or offer too much information. By integrating both approaches, the idea is to find a compromise between accuracy and efficiency (Fig. 9).^308^

## Network Analysis Software

- NetworkX.^309^ Python library used for the creation, manipulation, and study of the structure, dynamics, and functions of complex networks. This allows the creation of networks with different algorithms, evaluation of a large set of standard metrics, and finally display the results in an easily understood way. Freeware. Available at networkx.github.io
- Cytoscape.^310,311^ Software specialized on the representation of networks, with some additional tools for the integration of biological data. It also provides some basic network analysis capabilities. Freeware. Available at www.cytoscape.org
- Gephi.^312^ Interactive visualisation and exploration platform. Freeware. Available at gephi.github.io
- Pajek.^312^ Software for representing complex networks, with some basic analysis capabilities. Freeware. Available at mrvar.fdv.uni-lj.si/pajek/
- VisANT.^314^ Software for the visual study of metabolic networks and pathways. Freeware. Available at visant.bu.edu
- IBM^®^ i2 Analyst's Notebook. Software for the integration of social data and network analysis. Commercial. Information at www-03.ibm.com/software/products/en/analysts-notebook
- SAS^®^ Social Network Analysis. Software for the analysis of social networks. Commercial. Information at support.sas.com/software/products/sna/index.html

### networkAnalyst

Part of the same family of websites, including metaboAnalyst and microbiomeAnalyst, this website provides a visual analytics platform for meta-analysis of differentially expressed genes or proteins (www.networkanalyst.ca).^315,316^ It allows input of raw RNA-sequencing data, single or multiple gene expression tables, or pre-calculated lists of differentially regulated genes with expression values. The input is then compared with known interaction networks covering not only various protein–protein interactomes but also relationships between genes and miRNAs; TFs, drugs, or chemicals. By default, a first-order network is computed, which can also be switched to a second-order network to increase the number of interactors, or the zero-order network to decrease the number of nodes. If the complexity is too high, it can be reduced with filters on betweenness or degree. Another option is to calculate a minimum network, which comprises the least number of nodes that are required to link the input genes. The network can be downloaded in a Cytoscape-compatible SIF-format, but the standard routine is to visualize it within the web platform in an adjustable manner, including up- or downregulation of expression levels and different layouts, which can be saved in SVG-format. Moreover, and most importantly, the network can then be statistically compared with different databases such as KEGG, Reactome, gene ontologies, or TF motifs to obtain functional enrichment values. A module explorer can be applied to extract subnetworks with statistically elevated links, and these can be further analyzed for functional gene enrichments.

In case that the differential expression is computed on the *NetworkAnalyst* platform, gene clustering can be performed comprising heatmaps, principal-component analysis, or t-distributed stochastic neighbor embedding. Moreover, GSEA can be done and Venn- or Chord diagrams can be created for multiple comparisons.

### Network medicine

General terms are used to design applications of complex networks theory to medicine, and hence for the identification, prevention, and treatment of diseases.^84,317^ It is buttressed by the idea that elements constituting our bodies at all scales (e.g., from genes, to cells and organs) do not exist in an independent fashion, but are rather connected by a dense set of interdependencies. Understanding one disease, thus, goes beyond the simple analysis of one element. For further examples, see the Biological Networks section.

### Null models

In complex networks theory, a null model consists of a set of networks with some characteristics equal to the graph under study, while being random in all other aspects.^318^ The simplest case is, therefore, a set of completely random networks, that is, Erdős–Rényi graphs, which share the same number of nodes and links, but are otherwise completely random.

The main advantage provided by null models is that they allow breaking the coupling existing between different topological properties, and thus allow comparing networks with heterogeneous characteristics. To illustrate, the value of a given topological metric can be normalized with what is expected in the null model, thus helping to assess whether the observed value is special or, on the contrary, is the result of the other restrictions imposed in the model. The most simple solution involves the calculation of a Z-score, which indicates how many standard deviations the observed metric is from the (null model's) expected value.^202^

### Nvidia Clara

Nvidia Clara is a computational platform that gathers Compute Unified Device Architecture (CUDA) accelerated tools for medical imaging and genomics. The Software Development Kit (SDK) provides libraries for computing, visualization, and AI. The SDK allows the users to deploy their applications in any GPU platform they have access to. Within this platform, Nvidia Clara Medical Imaging provides tools for data annotation, training of AI models, and deployment in the case of medical imaging applications (e.g., computerized tomography, MRI, ultrasound, X-ray, and mammography). Adapting one of the included in the SDK pre-trained AI models with transfer learning accelerates the AI modeling, as less time and training data are used. On the other hand, the Nvidia Clara Genomics platform gathers CUDA accelerated tools for genomics sequencing and analysis. Biomedical examples of the use of Nvidia Clara include the segmentation of images of brain tumors,^319^ and gene sequencing.^320^

### Object-oriented modeling

For effective diagnosis and treatment of diseases we need to understand the dynamics of metabolism, including the metabolism of drugs. Here, the large-scale computational models that describe dynamics from the metabolic, gene regulatory, and signal transduction perspectives are of crucial value.^321^ Different modeling approaches are in use, including the object-oriented modeling. This technique is originally derived from machinery. Dymola (Dynamic Modeling Laboratory) has been developed by Dassault Systems, a branch of the Dassault group that also produces airplanes. Dymola sets the basics of object-oriented modeling of the biological systems even if its initial intention has been for use within automotive, aerospace, and robotics process. In Dymola, we can describe the entire multicomponent systems and in this manner represent the real world as good as possible.

The basics of object-oriented modeling is represented by a library of objects. An object is an element corresponding to components of mechanical, electrical, vehicle dynamics, etc., and also biological systems. In building the model, the objects from the library are moved by drag-and-drop and interactions between the model components are described by graphical connections that model the physical coupling of the components. The unique feature of object oriented modeling is that the models are intuitively organized to mimic the real physical or biological systems. In systems medicine, we can imagine that large macromolecules (genes, mRNAs, proteins including enzymes and TFs, etc.) are objects. The signaling pathways represent links or information that is transferred through connections between these objects.

Nowadays, Modelica is used as the most popular programming language for object-orienting modeling. The benefit of Modelica is that the users can create their own libraries. *BioChem* has been designed as a library for metabolic pathways^322^ that describes enzymatic reactions in different biochemical pathways. *SysBio* library^323^ was initially used to construct the *SteatoNet* model with multilayered regulation, including the transformation of genes to proteins and the transcriptional regulation.^324^ In addition, *SteatoNet* describes multiple tissues, that is, the liver and adipose tissue and their connections through the blood.

The beauty of object-oriented modeling is that the number of parameters that need to be incorporated into the model is small. We can, thus, avoid problems with parameter estimation or model overfitting. This is possible due to observation of the normalized steady state of the system's response, allowing modeling in the absence of parameters that describe the dynamics of the observed system. Another benefit of this type of modeling is the ability to incorporate specific data toward, that is, personalization. In this manner, the *LiverSex* has been produced as the first model describing the distinct liver metabolism of females and males.^325^

### Ontologies

Ontologies (also known as controlled vocabularies and semantic representation) can be defined as formal representations of knowledge in a certain domain, in an understandable way for people and computers.^326^ They are made of defined classes of entities, structured in hierarchy where concepts are connected with standardized relationships.^327^ In biomedical research, a great variety of ontologies have been developed to describe domain knowledge, for example, the Gene Ontology or the Disease ontology. BioPortal is a repository of biomedical ontologies, many of which can be openly reused. In addition, the open biomedical ontologies is an established platform developed for interoperability and shared principles between ontologies.^328^ The question of ontology relevance in the context of systems medicine has been particularly discussed. In fact, because of its intrinsic paradigm change, such ontologies must switch from a biological structure to a biological function architecture.^329^ Beyond the existing ontologies, the U.S. National Research Council proposed a new taxonomy for biology and medicine while taking into account the multiple aspects of basic science and clinical characteristics to define disease endotype.^330^ The development of phenotype-driven ontologies is also of great interest for the field.^331^ However, with the explosion of heterogeneous clinical data and scientific information, harmonization between scientific communities as well as their participation to computational resources are essential for the future of ontologies in translational research and precision medicine.^332^

### Parameter estimation

Mathematical models in systems biology and systems medicine have a structure that characterizes interactions between elements of the system. The next levels of detail are the parameters of interactions to quantify the intensity of interaction. Some of the model parameters can be measured or found in the literature, whereas information about others is missing. Parameter estimation^333^ can be used to estimate the unknown parameters by fitting of the model to the available experimental data. Usually, it is solved as a numerical optimization problem where the differences between measured data and model calculations have to be minimized, searching for the best combination of unknown parameter values. Parameter estimation can have several results:

- The model behavior fits the experimental data. It is not expected that model behavior would match each and every measurement, as they contain measurement errors and mathematical models are always simplifications of reality. Even in case of success, parameter identifiability should be checked (see the Parameter Identifiability section).
- The model behavior does not fit well to the experimental data. There can be several reasons: Model definition and range limitation of estimated parameters have to be checked. Another problem can be the selection of an inappropriate optimization method that leads to local minimum or stagnates.^334^
- The model cannot reproduce the expected type of behavior. This may be an indication that the structure of the model does not correspond to the system of interest; and that, without suitable changes in the model structure, a satisfactory behavior as well as an identification of parameters cannot be reached.

### Parameter identifiability

In case of successful parameter estimation, model parameters cannot be always trusted.^333^ It can happen that a value of a particular parameter is not important for particular experimental set-up and any value can produce an acceptable fit of model with experimental data. Another parameter unidentifiability reason can be structural unidentifiability,^335^ where the structure of the model in combination with experimental results does not allow the identification of particular parameters. For instance, if just summary flux of two parallel metabolic pathway branches is measured, parameters defining each particular flux cannot be identified.

### Parameter sensitivity analysis and uncertainty quantification

Parameter sensitivity analysis and uncertainty quantification are two important best practices when developing and simulating biological systems of interest. Parameter sensitivity analysis allows us to determine which parameters are sensitive to the input variations with the used constitutive laws.^336,337^ This analysis is commonly time-consuming due to the repetitive nature of the procedure. Moreover, the determination of a plausible perturbation value range is also a difficult issue. A relative percentage (e.g., ±10%) is usually used. Uncertainty quantification aims at modeling the uncertainties related to the system input values or variables and their propagation on the model outcomes through the used constitutive laws. A lot of data are commonly needed for uncertainty quantification. Data assumption could be performed with limited data samples, but the accuracy level is questionable. Precise and imprecise probabilities could be used to model uncertainties. Monte Carlo is a classic example of the uncertainty propagation method.^338^

### Permutation test

When we have to test between-group differences, for one or more values per subject, we can use a (nonparametric) permutation test to infer whether the difference between the two values is statistically significant or not. To do so, we need to generate random groups by shuffling the labels of the groups. The metric differences between the two resulting random groups are then used to create a reference distribution for each metric to reject or retain the null hypothesis that there are no differences between the groups. To ensure that the reference distribution is appropriate, we need to generate thousands of random groups. With 1000 random groups the smallest possible *p*-value is 10^−3^, whereas with 100,000 random groups the smallest possible *p*-value decreases up to 10^−5^. A practical way is to start with a not too large number of random groups, for instance 1000, and increase this number to a larger one if the *p*-value is small enough to be interesting. Because this calculation can be computationally demanding, sometimes parallel computing is needed. One way to avoid it is to use other techniques based on tail approximation, which obtain accurate *p*-values with a drastically reduced number of permutations.^339^ A typical case in which we will need to use the permutation test is when we are willing to test between-group differences in structural covariance analysis. In this case, we have the connectivity matrix at the group level and therefore the global connectivity measures are also at the group level. Testing differences between group level measures will require a permutation test.

### Phase transition

The original meaning of the term *phase transition* is to be found in statistical physics, and especially in thermodynamics. When one defines the *phase* of matter as a state in which it has uniformly physical properties, a phase transition occurs when that matter undergoes a transformation between two states. To illustrate, water and ice are two phases (respectively liquid and solid), and the transition between both of them (i.e., the freezing process) is a phase transition. The term is, nevertheless, also used in a more general sense, to indicate any transition between two homogeneous and easy identifiable conditions of a system. For instance, when deleting nodes from a complex networks to simulate an attack to the system, the initial connected status and the final disconnected one are two phases, with a transition in between them.^340^

**FIG. 10.**
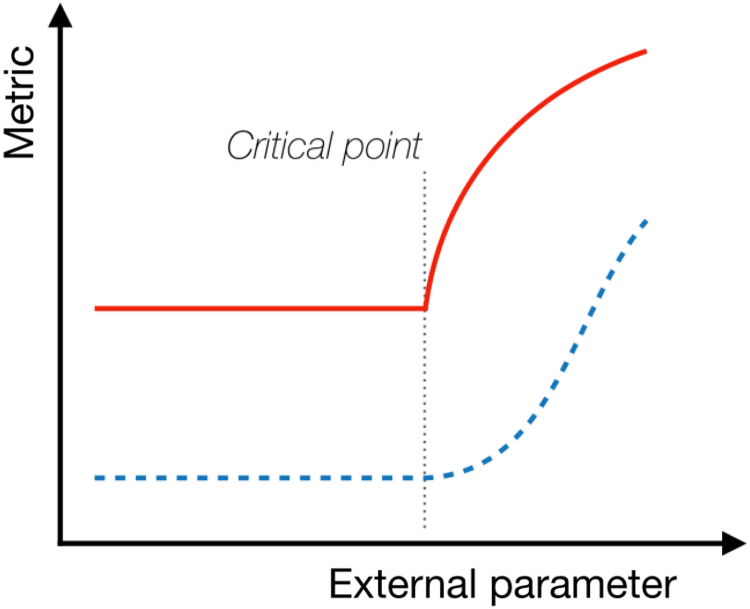
Example of two phase transitions, a first-order (red solid line) and a second-order one (dashed blue line).

Suppose one analyzes the evolution of some metric describing the system as a function of an external parameter; in the previous example, the former can be the connectedness of the network, which is studied as a function of the number of removed links. Two types of transitions can then occur:

- First-order phase transitions, which exhibit a discontinuity in the first derivative of the metric (solid red line of Fig. 10). This implies that the system has an abrupt reaction to the change in the external parameter.
- Second-order phase transitions are continuous in the first derivative, but they usually exhibit discontinuity in a second derivative (dashed blue line of Fig. 10). The response of the system is, therefore, smoother than in the previous case.

### Physiome

Physiome is a multiscale approach aiming at functionally synthesizing models at different levels, and at understanding human physiology based on computational models.^341^ Standardization of models has been part of this effort, and an important number of models is now available in the physiome repository (https://models.physiomeproject.org/welcome).

A flagship project has been the cardiovascular physiome, which aimed at using integrative multiscale modeling and linking the whole heart function with small-scale systems and phenomena (e.g., ion channel mutations, ischaemic tissue, drug toxicity, biochemical pathways), always with an eye toward providing tools for the clinician to investigate hypotheses and interpret experimental data. Within the physiome paradigm, the virtual physiological human (https://www.vph-institute.org/) has been a long-term initiative to embrace systems medicine at organism level, toward integrating all information available for each patient, and generating computer models to predict the patient's health evolution.

### Precision medicine

According to the HORIZON2020 Advisory Group (EU Health Ministers—December 2015), precision medicine is “a medical model using characterization of individual's phenotypes and genotypes (e.g., molecular profiling, medical imaging, lifestyle data) for tailoring the right therapeutic strategy for the right person at the right time, and/or to determine the predisposition to disease and/or to deliver timely and targeted prevention.” Precision medicine is then an approach to patient care that promotes the idea of doctors selecting most adequate treatments for patients based on a genetic understanding of their disease. This idea does not literally mean to create the drugs or medical devices that are specific for a patient, but divide the individuals into clusters (subpopulations) that differ in their susceptibility to a particular disease, biology, or prognosis of those diseases or response to specific treatments and select treatment based on that knowledge.^342^ Preventive or therapeutic interventions can then be concentrated on those who will actually benefit and save expenses on unnecessary treatments and side effects in patients who do not. An older synonym for precision medicine was “personalized medicine,” which was often misinterpreted as implying that unique treatments can be designed for each individual. As a result, the term “precision medicine” was created.^343^

### Probabilistic risk analysis

The PRA is aiming at quantitative measures for evaluation of the risk of system failures (e.g., supply of essential medicines within a health care system, availability of innovative drugs and active ingredients in the pharmaceutical sector, disruption of agri-food supply chains in natural disasters, security issues in the nuclear power industry), in which the common statistical analysis is very difficult or even impossible due to multiple and disparate issues (e.g., nonexistence of pertinent data, the system complexity, the uncertainty about consequences).^344^

The probabilistic risk is related with the probability distributions for the losses in a given time horizon, whereas the PRA methods also include event trees, fault trees, and Bayesian networks. The PRA approach typically considers: (i) identification of failure scenarios; (ii) computation of scenarios probabilities, by combination of events probabilities and the associated random variables distributions; and (iii) the evaluation of consequences, the extension and impacts of those scenarios. The data obtained in this way can then be used to feed a robust model with multiple goals, namely, by minimizing the expectance of system failure for a given budget (and/or for a given schedule), while verifying whether the probabilistic measures for risk failure are satisfactory.

The PRA is also strongly connected with other concepts of interest, such as Model robustness, Model Verification and Validation, Parameter Sensitivity Analysis and Uncertainty Quantification. Difficulties are usually associated with the scenarios definition, the selection of random variables distributions and events probabilities, as well as sparsity and high dimensionality.

### Quantitative systems pharmacology

Quantitative systems pharmacology (QSP) or systems pharmacology modeling is a computational and mathematical modeling approach that simulates the mechanistic effects of drug effectiveness.^345^ The QSP combines PK/pharmacodynamic (PD) modeling with systems biology and systems engineering.^346,347^ It integrates drug pharmacology, physiology, mathematics, and biochemistry, and it accounts for drug liberation, absorption, disposition, metabolism, and excretion. The QSP, which is a type of *in silico* modeling, typically makes use of differential equations to model the dynamics of the drug interacting with the biological system. More recently, the QSP involves genomic, transcriptomic, metabolomics, and proteomic levels, as well as regulatory and epigenomic levels. The QSP is increasingly being used in pharmaceutical research and development to help guide the discovery and development of new treatments and therapies, and to extrapolate animal data to humans.^348–350^ This is in line with recent directions in stratified medicine or precision medicine, by which model parameters can be tuned to simulate specific biomedical type. The advancement in big data and data science is gradually forming an integral part of QSP, complementing its traditional mechanistic modeling.

### Random forest

In data mining, RFs are classification algorithms based on combining multiple DTs models. The underlying concept is that an ensemble of models, each one independently trained on a subset of the data and each one casting a vote about a particular instance, could yield a better result than a single model, especially in problems that are characterized by a large number of variables, with each one of them encoding very little information. Following this idea, RFs are created by merging multiple DT predictors, with each one trained by using a different subset of the initial data.^351^ Each tree in RF is grown as follows: (i) sample with replacement a given number of cases from the training set at random. This sample will be the training set for growing the tree; (ii) given *M* input variables, randomly select m≪M of them at each node, and choose the best one to split the node; and (iii) grow the tree with no pruning. Given one new instance, the final classification corresponds to the class voted by the majority of the trees. Although there is no strict rule about the optimal number of trees to be grown, studies suggest that little is gained by growing more than 1000 trees.^352^

The RFs have three significant advantages: First, they do not suffer from overfitting, and can thus be used in small data sets. Second, their computational cost is reduced, and they are very prone to parallelization (as each tree can be created in an independent process). Finally, they have been shown to outperform most known algorithms, in terms of accuracy.^353^ On the negative side, it is worth noting that the number of trees in the model must be selected by the researcher, and that no clear rules are available to guide this process.

### Random graphs

Random graphs are graphs, or networks, that are artificially constructed by creating links between nodes according to a given probability distribution.^354,355^ As such, they do not correspond to any real-world system; but they instead provide a tool for answering specific questions about how some properties may appear. Due to the lack of any predefined structure, except for those naturally arising from the defined probability distribution, random graphs are well suited to be used as null models.

### Scale-free networks

A scale-free network is any complex network whose degree distribution approximatively follows a power law; in other words, the fraction of nodes with degree *k* goes as $P\left(k\right)\approx k^{-\gamma }$, with γ being a parameter usually in the range (2–3). Many real-world networks, including biological ones,^356,357^ have been found to be scale-free to some degree,^358,359^ although no consensus still exists on the best way of statistically testing such a property.^360^

Scale-free networks are of relevance for different reasons.

First of all, the degree distribution implies that most nodes have very few connections, whereas a (statistically significant) high number of them concentrate on the majority of the links; these latter ones are, thus, more important for the functioning of the network, or more central, and are usually called “hub.”

Second, the structure induced by scale-freeness implies a great resilience against random disruptions; note that, if a node is deleted at random, there is a high probability for that node to be secondary and weakly connected. On the other hand, a targeted attack can do much damage, as it can target a node of very high centrality.^361,362^

Finally, several models have been proposed to explain the appearance of scale-free networks^363–366^; and, more generally, the presence of such structure can point toward the existence of some generative processes.

### Simulated annealing

Simulated annealing (SA) is a form of optimization that is used to approximate global optimization in a large search space. This method is used in discrete space, where finding an approximate global optimum is more important than finding a precise local optimum in a fixed amount of time. In these situations, SA is often preferable to methods such as gradient descent. It is especially useful in finding global optima when large numbers of local optima are present. The SA uses the objective function of an optimization problem instead of the energy of material. Implementation of the SA consists of hill-climbing and picking a random move, instead of the best move. If the selected move improves the solution, it is accepted, and when not, it moves with a probability of less than 1. The value of probability decreases exponentially with the amount of how much the solution is worsened.^367,368^ Beyond general optimization problems (see, for instance, Refs.^369–371^), SA has extensively been used for segmenting medical images.^372,373^

### Small-world network

The theory of small-world networks^374^ is based on the observation of biologic or complex systems that can be represented by using graphical models. The specific graph shows especial characteristics, such as having a high clustering of its elements, and a very fast association between any two different nodes that can be inferred by following the shortest path between the nodes through the graph connections.

The formulation of small-world networks was inspired by the idea that the “degree of separation” or distance between two different (unfamiliar) people on the Earth is about five.^375^ Not only social networks have been observed to follow this pattern, but networks of collaborators, complex systems, and brain networks also follow this interesting rule.

**FIG. 11.**
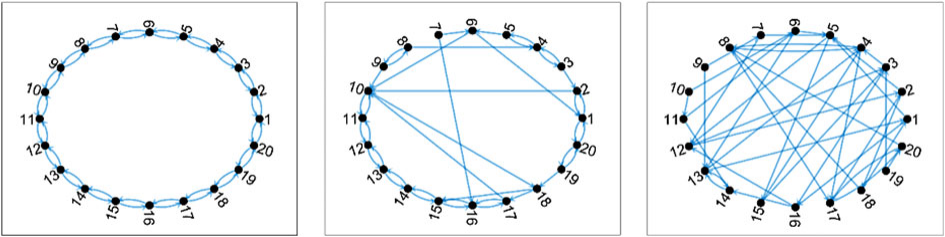
Example of the creation of a small-world network.

A small-world network can be also explained as the transition from random or chaotic systems to highly regular or structured ones. For example, in a regular lattice network, where the nodes only have connections to the closest or adjacent nodes, it can be observed that by disconnecting and randomly reconnecting the nodes, the average distance between any two nodes in the network rapidly decays whereas maintaining the local network of closest nodes only decays slightly in density (clustering coefficient). In neural networks, this property of small-worldness can be seen as critical to maintain a fast integration among distant neural populations to process information efficiently, whereas the different tokens of information are locally processed in highly dense local networks (Fig. 11).

### Smoothed-particle hydrodynamics

Smoothed-particle hydrodynamics (SPH) is a computational method that is used for simulating the mechanics of continuum media, such as solid mechanics and fluid flows.^376^ Many fields of research have employed the SPH method, such as engineering, astrophysics, ballistics, volcanology, and oceanography.^377–379^ It is a meshfree Lagrangian method, meaning there is no division of domains of interest in the form of mesh (see Finite Element Method and Finite Volume Method sections), but rather the coordinates move with the fluid. In such a way, the resolution of the method can easily be adjusted with respect to variables such as density. Here, the computational domain is discretized by a finite set of interpolating points (particles), with invariant coordinates in the material frame. Each SPH particle represents a finite mass of the discretized continuum and carries the information about all physical variables that are evaluated at their positions. Interpolating (smoothing) function and its derivatives at surrounding particles are used to evaluate the function values and their derivatives at a specific particle.^380^ The SPH has been used, for instance, to model therapeutic solutions aimed at helping heart muscle to regenerate after an injury.^381^

### Solid–fluid interaction

Solid–fluid interaction is a numerical approach that is used to model phenomena that involve both the surrounding fluid and immersed solid objects. Using this approach, both domains are simulated concurrently, and they form a coupled mechanical system. The fluid is acting on the solid object via external forces and causes the motion and deformation of the deformable solid and *vice versa*—the solid is opposing the deformation and the influence of fluid and in this way alters the fluid flow. Solid–fluid interaction techniques have been applied, for instance, in modeling the deployment of the stent within the stenotic artery with a deformable arterial wall^382^; in simulating the behavior of deformable cells within a fluid flow^383,384^; and in providing insight into the benefits of different treatment alternatives in a case of type B aortic dissection.^385^

### Statistical bioinformatics

The application of statistical techniques is mainly to large sets of biomedical data—mainly genomics data, but recently this has evolved to include any type of -omics data. For more information, refer to Refs.^386–389^

### Statistical networks

One of the properties of a system is that it consists of interacting components at different levels. Creating a corresponding network may be based on biology (see the Biological Networks section) or may be based on analytical arguments, or both. Statistical epistasis networks belong among the simplest examples of such networks, in which nodes refer to units of analysis and edges are formed via a notion of statistical significance. They have become popular tools in genome-wide association interaction studies to highlight higher-order interactions in typically underpowered studies.^390^ In general, the major challenge with statistical networks is to assess and minimize statistical artefacts that may hamper network-derived biological conclusion-drawing.^391^

### Support vector machine

Binary linear classifiers are based on the identification of hyperplanes in the feature space, dividing the training instances in two groups according to the training label. The model is trained by first constructing a feature space, that is, a hyper-space defined by the features available in the data set, which must always be numerical. Records are mapped into this space, and the best linear separation between them is then calculated. The best separation is achieved by the hyperplane that has the largest distance to the nearest training-data point of any class, as this minimizes the error. Modified versions of SVMs have been developed to tackle different problems, including regression problems,^392^ or the use of different kernels (i.e., distance functions) to obtain nonlinear models.^393^ Among SVMs, disadvantages are a high computational cost, and the complexity of dealing with classifications with multiple labels. For more details, refer to Refs.^394,395^

### Surrogate model

Surrogate model is an engineering method that is used when an outcome of interest cannot be easily directly measured, and instead, a model of the outcome is used. In many real-world problems, one simulation can take from minutes, to hours and even days to finish the calculation. Therefore, sometimes design optimization, sensitivity analysis, and what-if analysis are impossible to investigate, since that would mean running thousands or even millions of simulations. Surrogate models, also known as metamodels, are compact, scalable analytic models that approximate the multivariate input/output behavior of complex systems, based on only a limited set of computationally expensive simulations. In such a way, surrogate models actually mimic the complex behavior of the simulation model, and they are applied in design automation, parametric studies, design space exploration, optimization, and sensitivity analysis. Other synonyms for surrogate models are response surface models, emulators, auxiliary models, repro-models, metamodels, etc.^396^

### Systems biology

Systems biology is the field devoted to the computational and mathematical modeling of complex biological systems.^397–399^ It focuses on the relationships between the components of a biological system, and how these relationships give rise to its global function and behavior. This is opposed to a reductionist paradigm.

### Systems bioinformatics

A new approach to the analysis of biomedical data is based on the application of a systems biology perspective. This includes, on one hand, a top-down view, with bioinformatics methods being used to extract and analyze information from “omics” data generated through high-throughput techniques,^400^ eventually integrating omics data coming from different sources.^401–403^ On the other hand, this is complemented with a bottom-up approach, where information from molecular cells and tissues, alongside mathematical models, is used to elucidate the function and dynamic behavior of cells, organs, and organisms.

### Systems dynamics

Systems dynamics or dynamical systems is a mathematical method or modeling approach for understanding the behavior of complex systems, with their states evolving over time. This is used in *in silico* modeling of biomedical systems. For instance, biochemical reactions (using mass action law), intracellular signaling pathways, activity of excitable/nerve cells and their networks, biological rhythms, cancer development, and population dynamics can be described by dynamical systems.^404–408^

A system often consists of a set of interacting elements or components that forms a larger component or entity. Understanding the latter's behavior is often not immediately clear just based on the elements or building blocks, but through the analysis of the interactions leading to “emergent” dynamical behavior. The analysis could be performed analytically (especially for simpler systems) or computationally by using various numerical methods. Often, the stability of the system is also evaluated analytically or computationally either locally, for example, around some steady state, or globally. Software are often used for numerical computation. The popular ones include XPPAUT (C programming based)^409^ and MATCONT (MATLAB programming based).^410^

The elements or interactions can be linear or nonlinear. The interactions can be instantaneous or time-delayed. The system can be deterministic or stochastic (i.e., in the presence of noise). Suppose a system's state variable is described by a vector *x*, and the environment of system is described by parameters *a*, the evolution mechanism of dynamical systems can be continuous (behaving continuously over time) and described by a group of differential equations,

$$\frac{dx}{dt}=f\left(x,a,t\right),$$

or discrete (behaving over discrete time points) and described by difference equations,

$$x\left(t+1\right)=f\left[x\left(t\right),a\right],$$

or described by symbolic dynamics, that is, mathematical function mappings^408^

$$f:x\left(t\right)\to x\left(t+1\right).$$

Often but not necessary, nonlinearity in the system can lead to highly nontrivial emergent dynamics. For instance, varying some parameter around its critical value can dramatically change the behavior of the system. This is termed bifurcation^411^ or phase transition, and it is linked to the Catastrophe Theory.^412^ Some other topics related to systems dynamics or dynamical systems theory include the Chaos Theory.^408^

### Systems engineering

Systems Engineering is a multi/transdisciplinary field devoted to the engineering and engineering management of very large and complex socio-technical systems. It addresses all the elements within a system; their individual properties and inter-relations are considered and integrated in a holistic approach, through a combination of relationships to jointly perform a useful function as a whole. Systems Engineering combines Engineering with Management, Finance, Economics, Pure/Exact, and Social Sciences, in a way to adequately design, develop, and implement the large and complex systems that are so important nowadays. It is typically used to manage the inherent complexity of societal problems, for example, either in spacecraft design or in combination with PK/PD modeling and Systems Biology.^346,347^ In this way, the Systems Engineering approaches are delimited within the Systems Theory framework.^413^

### Systems medicine

Systems medicine is an interdisciplinary field of study that looks at the human body as a system, composed of interacting parts, and further integrated into an environment. It considers that these complex relationships exist on multiple levels, and that they have to be understood in light of a patient's genomics, behavior, and environment. As such, it integrates contributions from multiple research fields, including medicine, systems biology, statistics, modeling and simulation, and data science. The earliest uses of the term “systems medicine” appeared in 1992, in two articles independently published by Zeng^3^ and Kamada.^4^

As the name suggests, “systems medicine” represents the convergence of two main fields:

- Systems biology, the field of study that focuses on complex interactions within biological systems, using a holistic approach.
- Medicine, as it presents a clear focus toward medical research and medical practice. As such, systems medicine aims at having tangible benefits for the patients, with the identification of those elements that are critical for influencing the course of the system (i.e., medical conditions).

Among its objectives, it is worth highlighting:

- Systems medicine is not systems biology just in one species, but similar to the distinction between “medicine” and “biology” systems medicine needs to have an objective to achieve patient benefit, by either better or earlier diagnosis and therapy.
- Systems medicine questions and replaces the current concept of medicine, which is largely built on organ-based subfields and symptom-based disease definitions, toward a holistic-defining diseases at a mechanistic level.
- Systems medicine defines (diagnostic and therapeutic) targets not any longer as single molecules but rather perturbed networks, which form subgraphs of the interactome.
- At the application side, systems medicine will lead to precision diagnostics and therapeutics.
- Some therapeutics/drugs will not need to be developed *de novo* but repurposed/repositioned.
- Use multilayer diagnostic tools.
- Thus, systems medicine will enable predictive, personalized, preventative, and participatory medicine.
- By increasing medical precision and efficacy, systems medicine ideally addresses the financial pressures on all health care providers and enables the ultimate move from an input medicine to an output medicine (see recent World Economic Forum Davos).

### System of systems

Systems of Systems can be represented as large-scale, complex, and distributed systems. System of Systems concept is described in terms of “Maier's criteria”^414^: operational and managerial independence, distribution, and emergent behavior as a result of component behavior and evolutionary development. System of Systems principles can be applied in integrating health management, medical diagnosis, and medical support systems.^415^

### Standards

The word “standard” has several different definitions. In general metrology, a standard is a reference that is used to calibrate measurements, whereas in the systems biology field, standards have been developed through standardization initiatives (e.g., ISO, COMBINE^416^) to format and describe data and models, for exchange and understanding between scientific communities. Three types of standards have been considered^417^: standard formats for representing data and models; standard metadata for describing types of data and models; and controlled vocabularies and ontologies to provide a common vocabulary.

### Structural covariance networks

A technique is used to reconstruct complex networks representations of brain cortical regions. The network is defined such that nodes represent brain regions, and link the Pearson's correlation of CT or volume between pairs of regions, as yielded by magnetic resonance data (MRI).^418,419^ Structural covariance between regions can be used to construct the so-called structural covariance networks. Several studies have been conducted in which structural covariance networks have been analyzed in healthy subjects,^420,421^ and in groups of patients with disorders such as autism, attention deficit hyperactivity disorder, schizophrenia, or Alzheimer's disease,^422–425^ or to assess the differences between gifted children and controls.^426^ Since the SCN is at the group level, (structural) connectivity parameters are also at the group level and a permutation test will be needed to infer differences between measures. See also the Morphometric Similarity Networks section.

### Time-evolving networks

One major problem that was found while studying time-evolving systems through complex networks was that edges may not continuously be active. To illustrate this, let us consider the network of contacts between inpatients of a hospital, which may be used to model the propagation of infectious diseases. First, two people may be connected by a link even if they have been in the same room for a short time window; thus, the probability of contagious should not be binarized. Second, the sequence of contacts is also important: If a person met patient *A* and later patient *B*, a disease cannot spread from *B* to *A*. The solution was the development of the concept of time-evolving, or temporal, networks, in which a collection of networks represent the status of the system as it evolves through time.^427,428^

### Time-scale separation

Dynamic mathematical models can be simplified by using the time-scale separation approach: If part of a system operates sufficiently fast compared with the rest of the system, it may be assumed to have reached a steady state.^429^ This allows the elimination of fastest components from the model, lumping them with slower components as they determine the speed of a systems reaction. This approach can be very efficient in multiscale modeling, where dynamics of very different processes are merged. Time-scale separation is applied for the modeling of vector-borne diseases, where human host epidemiology is much slower than the transmission of vector from human to human by mosquitos: Only the human time scale is investigated assuming that human–human transmission happens instantly.^430^ Time-scale separation can be used to simplify modeling of biochemical processes at the cellular physiology level.^431^

### Variation partitioning

Also called “commonality analysis,” a technique aimed at quantifying the part of the observed variation, that is, the shared consequence of two (or more) explanatory variables. It was initially introduced in 1992 by Borcard et al. in ecology,^432^ and it has since seen some limited applications in medicine.^433,434^

### Virtual physiological human

See the Physiome section.

## Notes

**Cite this article as:** Zanin M, Aitya NAA, Basilio J, Baumbach J, Benis A, Behera CK, Bucholc M, Castiglione F, Chouvarda I, Comte B, Dao T-T, Ding X, Pujos-Guillot E, Filipovic N, Finn DP, Glass DH, Harel N, Iesmantas T, Ivanoska I, Joshi A, Boudjeltia KZ, Kaoui B, Kaur D, Maguire LP, McClean PL, McCombe N, Miranda JLd, Moisescu MA, Pappalardo F, Polster A, Prasad G, Rozman D, Sacala I, Sanchez-Bornot JM, Schmid JA, Sharp T, Solé-Casals J, Spiwok V, Spyrou GM, Stalidzans E, Stres B, Sustersic T, Symeonidis I, Tieri P, Todd S, Van Steen K, Veneva M, Wang D-H, Wang H, Wang H, Watterson S, Wong-Lin KF, Yang S, Zou X, Schmidt HHHW (2021) An early stage researcher's primer on systems medicine terminology, *Network and Systems Medicine* 4:1, 2–50, DOI: 10.1089/nsm.2020.0003.

## Notes

^*^ We plan this glossary to be updated in the future; we will, therefore, welcome any suggestion coming from readers.
